# Indicators for Monitoring Recovery From Surgery to Discharge Using Accelerometer in Patients With Esophageal Cancer

**DOI:** 10.1002/wjs.70472

**Published:** 2026-07-02

**Authors:** Takahiro Yamane, Yoshikazu Kimura, Manami Tetsutani, Shota Yamamura, Airu Ito, Moe Tanuma, Mina Honjoh, Yoshiko Moriwaki, Kazuhiro Noma, Shunsuke Tanabe, Naoaki Maeda, Yoshikazu Matsuoka, Mizuki Morita, Hiroshi Morimatsu

**Affiliations:** ^1^ Department of Biomedical Informatics Okayama University Graduate School of Interdisciplinary Science and Engineering in Health Systems Kita‐ku Okayama Japan; ^2^ Department of Anesthesiology and Resuscitology Okayama University Graduate School of Medicine, Dentistry, and Pharmaceutical Sciences Kita‐ku Okayama Japan; ^3^ Faculty of Health Sciences Okayama University Medical School Kita‐ku Okayama Japan; ^4^ Department of Gastroenterological Surgery Okayama University Graduate School of Medicine Dentistry and Pharmaceutical Sciences Kita‐ku Okayama Japan

**Keywords:** actigraphy, esophageal cancer, monitoring indicator, physical activity, postoperative recovery, surgery‐to‐discharge

## Abstract

Esophageal cancer is a highly invasive malignancy necessitating esophagectomy, which is associated with considerable postoperative morbidity and prolonged hospitalization. Enhanced Recovery After Surgery (ERAS) protocols recommend early mobilization to facilitate recovery; however, objectively assessing physical activity during hospitalization remains challenging. Traditional methods, such as the 6‐min walk test or patient‐reported questionnaires can be burdensome, or may not accurately reflect recovery. Accelerometer‐based measurement provides a low‐burden, objective approach, but previous studies focused on the immediate postoperative period or long after discharge, leaving the recovery trajectory from surgery to discharge underexplored.

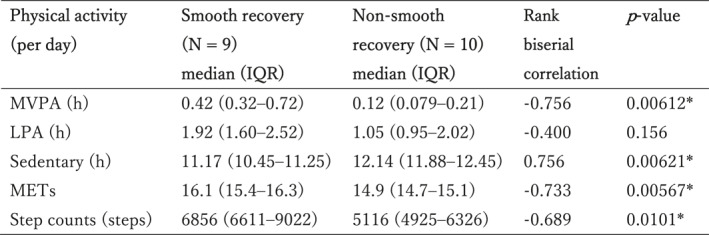

## 1 Introduction

Esophageal cancer is a highly invasive malignancy necessitating esophagectomy, which is associated with considerable postoperative morbidity and prolonged hospitalization [1]. Enhanced Recovery After Surgery (ERAS) protocols recommend early mobilization to facilitate recovery; however, objectively assessing physical activity during hospitalization remains challenging. Traditional methods, such as the 6‐min walk test [2] or patient‐reported questionnaires [3] can be burdensome, or may not accurately reflect recovery. Accelerometer‐based measurement provides a low‐burden, objective approach, but previous studies focused on the immediate postoperative period or long after discharge [4, 5, 6], leaving the recovery trajectory from surgery to discharge underexplored. This study aimed to identify the most effective accelerometer‐derived physical activity indicators—moderate‐to‐vigorous physical activity (MVPA), light‐intensity physical activity (LPA), sedentary behavior, metabolic equivalents of task (METs), and step counts—for evaluating recovery during hospitalization following esophageal cancer surgery, thereby providing a basis for improved monitoring and tailored care strategies.

## 2 Methods

This prospective observational study was conducted at Okayama University, Japan, between June 2023 and June 2024. Adult patients (≥ 18 years) undergoing esophageal cancer resection were enrolled. Individuals unable to wear the device, complete questionnaires, or those participating in other prospective studies were excluded. Baseline and surgical characteristics were extracted from medical records after participants provided informed consent.

Physical activity was measured using a wrist‐worn accelerometer (ActiGraph wGT3X‐BT, ActiGraph, Pensacola, FL, USA), worn continuously on the nondominant wrist for 14 days postoperatively, except during water exposure or imaging. Because the accelerometer was removed during postoperative day (POD) 14, only POD1 to POD13 provided complete 24‐h data, and analyses were therefore restricted to this period. Data were included if ≥ 4 valid days (≥ 10 h/day) were recorded [7]. Activity between 8 a.m. and 10 p.m. was analyzed as daytime activity, and hourly values within this period were summed to obtain the daily value. Indicators included MVPA, LPA, sedentary behavior, METs, and step counts. ActiGraph setting details are provided in Supporting Information S1: Online Resource 1.

Recovery stages (0–6) were obtained from rehabilitation records, reflecting stepwise functional goals applied in clinical practice. Stage 6 (independent ambulation to the rehabilitation room) was regarded as most advanced. Details are available in Supporting Information S2: Table S1 in Online Resource 2. Recovery trajectories from POD1 to POD13 were constructed using these recovery stages and used to classify patients according to recovery pattern. Patients whose trajectory monotonically increased and reached recovery stage 6 during POD1–13 were classified as the “smooth recovery” group. Here, “monotonic increase” was defined as the rehabilitation stage remaining unchanged or progressing over time after surgery, although a single instance of a one‐stage decline was permitted. Patients who did not reach recovery stage 6 during POD1–13 were classified as the “non‐smooth recovery” group. One patient who reached recovery stage 6 but showed two declines of two or more stages was classified as belonging to the “neither group.” Further details are provided in Supporting Information S2: Online Resource 2.

For each patient, trajectories of postoperative activity indicators (MVPA, LPA, sedentary behavior, METs, and steps) were plotted from POD 1 to POD 13, with linear regression lines fitted to these trajectories. Trajectories of recovery and physical activity, together with fitted linear regression lines, are provided in Supporting Information S2: Figure S1–S3 in Online Resource 2.

Statistical analyses were performed using EZR version 1.61 (Saitama Medical Center, Jichi Medical University, Saitama, Japan) [8]. For group comparisons, the median daily values of each physical activity indicator during POD1–13 were calculated for each patient and compared using the Mann–Whitney *U*‐test, with effect sizes expressed as rank‐biserial correlations [9]. Spearman's correlation coefficients were used to evaluate the associations of length of postoperative hospital stay with both the slopes of the linear regression lines and the median values of the five activity indicators. A two‐sided *P* ≤ 0.05 was considered significant.

## 3 Results

Twenty‐one patients who met the inclusion criteria and consented to participate were included. One participant was excluded due to surgical procedure changes, and another was excluded from the Mann–Whitney *U*‐test because of categorization into the “neither group”. Two participants declined to wear the monitoring device during the observation period, and their partial data were included. Participant characteristics and perioperative data are presented in Supporting Information S3: Online Resource 3.

The classification of recovery trajectories derived from rehabilitation records was examined in relation to MVPA trajectory patterns, because functional recovery accompanying rehabilitation progression was expected to be associated with increases in physical activity. This correspondence was supported by visual inspection of individual trajectories and by between‐group comparisons of patient‐level median MVPA from POD1 to POD13. Patients in the “smooth recovery” group exhibited increasing MVPA, whereas those in the “non‐smooth recovery” group maintained low levels (Supporting Information S2: Online Resource 2; Figure S2 (a, b)). METs demonstrated similar patterns. Individual trajectories are presented in Supporting Information S4: Online Resource 4. Each patient's median MVPA, sedentary behavior, METs, and step counts differed significantly between “smooth” and “non‐smooth” recovery groups (Table 1).

**TABLE 1:** Physical activity and recovery.

Physical activity (per day)	Smooth recovery (*N* = 9) median (IQR)	Non‐smooth recovery (*N* = 10) median (IQR)	Rank biserial correlation	*p*‐value
MVPA (h)	0.42 (0.32–0.72)	0.12 (0.079–0.21)	−0.756	0.00612*
LPA (h)	1.92 (1.60–2.52)	1.05 (0.95–2.02)	−0.400	0.156
Sedentary (h)	11.17 (10.45–11.25)	12.14 (11.88–12.45)	0.756	0.00621*
METs	16.1 (15.4–16.3)	14.9 (14.7–15.1)	−0.733	0.00567*
Step counts (steps)	6856 (6611–9022)	5116 (4925–6326)	−0.689	0.0101*

Abbreviations: IQR, interquartile range; LPA, light‐intensity physical activity; METs, metabolic equivalents of task; MVPA, moderate‐to‐vigorous intensity physical activity.

^*^ Significantly different (*p* < 0.05).

No statistically significant differences were observed in each patient's median values of the accelerometer‐derived physical activity measures between patients with and without postoperative complications (Table 2). The categories and types of postoperative complications and number of patients in whom they occurred are presented in Supporting Information S5: Online Resource 5.

**TABLE 2:** Physical activity and postoperative complications.

Physical activity (per day)	Postoperative complications (Absent) (*N* = 7) median (IQR)	Postoperative complications (Present) (*N* = 13) median (IQR)	Rank biserial correlation	*p*‐value
MVPA (h)	0.27 (0.18–0.64)	0.23 (0.12–0.42)	0.022	0.968
LPA (h)	1.92 (1.61–2.39)	1.44 (0.95–2.18)	0.319	0.275
Sedentary (h)	11.20 (10.89–11.58)	11.57 (10.32–12.15)	−0.132	0.663
METs	15.50 (15.06–16.15)	15.33 (14.93–16.25)	0.011	1.000
Step counts (steps)	6512 (5565–6827)	6611 (5172–7991)	−0.121	0.699

Abbreviations: IQR, interquartile range; LPA, light‐intensity physical activity; METs, metabolic equivalents of task; MVPA, moderate‐to‐vigorous intensity physical activity.

Analyses were performed to examine the relationship between MVPA and recovery stage. When MVPA increased by ≥ 0.5 h over the preceding 2 days, or when MVPA on the previous day was ≥ 1 h, the recovery stage on the following day was generally stage 5 or higher (Supporting Information S6: Online Resource 6; Figure S5 (a, b)). Similar patterns were observed for other physical activity indicators (Supporting Information S6: Online Resource 6; Figure S5 (c–j)).

Significant correlations were observed between the slopes of all five activity indicators and the length of postoperative hospital stay, and also between the median values of MVPA, LPA, sedentary behavior, and METs and the length of postoperative hospital stay (Table 3). Among the 10 patients with an MVPA slope < 0.05 or a median MVPA < 0.25 h, 8 had a postoperative hospital stay longer than the study median of 27.5 days (Supporting Information S7: Online Resource 7; Figure S6 (a, b)). Similar patterns in relation to prolonged postoperative hospital stay were observed for the other physical activity indicators (Supporting Information S7: Online Resource 7; Figure S6 (c–j)).

**TABLE 3:** Correlation coefficients between slope/median and LOS.

	Slope and LOS		Median and LOS	
	Coefficient	*p*‐value	Coefficient	*p*‐value
MVPA	−0.568	0.00896*	−0.501	0.0246*
LPA	−0.553	0.0114*	−0.500	0.0246*
Sedentary	0.548	0.0124*	0.561	0.0101*
METs	−0.579	0.0075*	−0.495	0.0264*
Step counts	−0.474	0.0347*	−0.431	0.0578

Abbreviations: LOS, length of postoperative hospital stay; LPA, light‐intensity physical activity; METs, metabolic equivalents of task; MVPA, moderate‐to‐vigorous intensity physical activity.

^*^ Significantly associated (*p* < 0.05).

## 4 Discussion

This study identified MVPA, sedentary behavior, and METs as the most effective indicators for assessing recovery status during postoperative hospitalization in patients undergoing esophageal cancer surgery. These indicators were significantly correlated with length of postoperative hospital stay and differed consistently between the smooth and non‐smooth recovery groups. These findings suggest that increased physical activity, as measured by accelerometers, reflects faster recovery and may provide a simple, objective method for monitoring patients during hospitalization. In particular, among daytime activity indicators, MVPA and METs may be preferable recovery metrics compared with LPA or step counts, which may be less reflective of recovery status. Thus, tracking daily increases in MVPA or METs, alongside reductions in sedentary time, may enable timely and individualized rehabilitation interventions. Our results are consistent with those of previous reports indicating that low early postoperative activity is associated with longer hospital stays [10].

In daily clinical practice, accelerometer‐derived MVPA could serve as a simple, objective signal of postoperative recovery. Patients whose MVPA increased by ≥ 0.5 h over the preceding 2 days, or who achieved ≥ 1 h of MVPA on the previous day, may have regained sufficient mobility to walk independently to the toilet or participate in rehabilitation exercises in the rehabilitation room (Supporting Information S6: Online Resource 6; Figure S5 (a, b)). By POD14, activity data collected from POD1 to POD13 may also help identify patients at risk for prolonged postoperative hospital stay. In particular, patients with an MVPA trajectory slope < 0.05 or a median MVPA < 0.25 h during POD1–13 often required longer hospitalization (Supporting Information S7: Online Resource 7; Figure S6 (a, b)). These preliminary thresholds suggest that wearable accelerometer data may help clinicians adjust the timing, intensity, and content of rehabilitation according to each patient's recovery status.

This study has some limitations. The sample size was small, no formal power analysis was conducted, and it was a single‐center study with a homogeneous patient population undergoing thoracoscopic/laparoscopic esophagectomy. Recovery status was assessed based on rehabilitation records, which may include subjective elements. Nevertheless, as a pilot study, these findings justify larger, multicenter trials aimed at standardizing accelerometer‐based recovery monitoring.

## Author Contributions

**Takahiro Yamane:** data curation, formal analysis, investigation, writing – original draft. **Yoshikazu Kimura:** data curation, investigation, writing – review and editing. **Manami Tetsutani:** data curation, formal analysis, investigation, visualization. **Shota Yamamura:** data curation, formal analysis, investigation, visualization. **Airu Ito:** data curation, investigation. **Moe Tanuma:** formal analysis, visualization. **Mina Honjoh:** formal analysis, validation. **Yoshiko Moriwaki:** data curation, investigation, project administration. **Kazuhiro Noma:** investigation, resources, writing – review and editing. **Shunsuke Tanabe:** investigation, resources, writing – review and editing. **Naoaki Maeda:** investigation, resources, writing – review and editing. **Yoshikazu Matsuoka:** conceptualization, methodology, writing – review and editing. **Mizuki Morita:** conceptualization, methodology, supervision, writing – review and editing. **Hiroshi Morimatsu:** conceptualization, methodology, supervision, writing – review and editing.

## Funding

The authors have nothing to report.

## Ethics Statement

This study was conducted in accordance with the Declaration of Helsinki and was approved by the Ethics Committee of Okayama University (approval number K2302‐049).

## Consent

Informed consent was obtained from all participants included in the study.

## Conflicts of Interest

The authors declare no conflicts of interest.

## Supporting information


Supporting Information S1

Supporting Information S2

Supporting Information S3

Supporting Information S4

Supporting Information S5

Supporting Information S6

Supporting Information S7

## Data Availability

The data that support the findings of this study are available on request from the corresponding author. The data are not publicly available due to privacy or ethical restrictions.
