# Supplementary material for: Indicators for Monitoring Recovery From Surgery to Discharge Using Accelerometer in Patients With Esophageal Cancer
Source: World J Surg. 2026 Jul 2;50(8):2434–7. doi: 10.1002/wjs.70472 (PMC13460619; doi:10.1002/wjs.70472)
Supplement: Supplementary file 1 [file WJS-50-2434-s001.pdf]

T. Yamane, Y. Kimura, M. Tetsutani, et al., “Indicators for Monitoring Recovery From Surgery to Discharge Using Accelerometer in Patients With Esophageal Cancer,” *World Journal of Surgery* (2026): https://doi.org/10.1002/wjs.70472

Methods (Online Resource 1)

ActiGraph settings

The used cutoff counts per activity intensity level were sedentary behavior (≤1853 counts/min), light-intensity physical activity (LPA) (1854-3268 counts/min), and moderate-to-vigorous physical activity (MVPA) (≥3269 counts/min) with 90 Hz measurement epoch. These cut points were based on vector magnitude. They were defined referring to previous studies [1,2]; cut points for sedentary behavior and MVPA of elderly people wearing ActiGraph GT3X+ on the non-dominant wrist were determined with experiments. Since patients suffering from esophageal cancer are generally elderly people and the patients wore the device on their non-dominant wrist, we applied these cut points to this study. The ActiGraph recorded data was downloaded in 60 second epochs of all three axes using ActiLife software version 6.13.4 (ActiGraph, Pensacola, FL, USA). To identify non-wear time, the algorithm of Choi et al. was used [3] because it was recommended for elderly people [4]. This algorithm defines non-wear times as periods of consecutive 0-counts for the duration of 90 min. Low filter extension was applied to capture slow activities of elderly people [4].

References

1. Koster A, Shiroma EJ, Caserotti P, et al (2016) Comparison of Sedentary Estimates between activPAL and Hip- and Wrist-Worn ActiGraph. *Med Sci Sports Exerc* 48:1514–1522. https://doi.org/10.1249/MSS.0000000000000924.
2. Bammann K, Thomson NK, Albrecht BM, et al (2021) Generation and validation of ActiGraph GT3X+ accelerometer cut-points for assessing physical activity intensity in older adults. The OUTDOOR ACTIVE validation study. *PLoS One* 16:1–12. https://doi.org/10.1371/journal.pone.0252615.
3. Choi L, Liu Z, Matthews CE, Buchowski MS (2011) Validation of accelerometer wear and

nonwear time classification algorithm. *Med Sci Sports Exerc* 43:357–364. https:// doi.org/10.1249/MSS.0b013e3181ed61a3.

4. Migueles JH, Cadenas-Sanchez C, Ekelund U, et al (2017) Accelerometer Data Collection and Processing Criteria to Assess Physical Activity and Other Outcomes: A Systematic Review and Practical Considerations. *Sports Med* 47:1821–1845. https://doi.org/10.1007/s40279-017-0716-0.
