# Supplementary material for: Indicators for Monitoring Recovery From Surgery to Discharge Using Accelerometer in Patients With Esophageal Cancer
Source: World J Surg. 2026 Jul 2;50(8):2434–7. doi: 10.1002/wjs.70472 (PMC13460619; doi:10.1002/wjs.70472)
Supplement: Supplementary file 2 [file WJS-50-2434-s002.pdf]

Online Resource 7

Indicators for Monitoring Recovery From Surgery to Discharge Using Accelerometer in Patients With Esophageal Cancer

Takahiro Yamane[1]*, Yoshikazu Kimura[2]*, Manami Tetsutani[3], Shota Yamamura[3], Airu Ito[3], Moe Tanuma[3], Mina Honjoh[3], Yoshiko Moriwaki[2], Kazuhiro Noma[4], Shunsuke Tanabe[4], Naoaki Maeda[4], Yoshikazu Matsuoka[2], Mizuki Morita[1,3]†, Hiroshi Morimatsu[2]

[1] Department of Biomedical Informatics, Okayama University Graduate School of Interdisciplinary Science and Engineering in Health Systems
[2] Department of Anesthesiology and Resuscitology, Okayama University Graduate School of Medicine, Dentistry, and Pharmaceutical Sciences
[3] Faculty of Health Sciences, Okayama University Medical School
[4] Department of Gastroenterological Surgery, Okayama University Graduate School of Medicine, Dentistry and Pharmaceutical Sciences,
* These authors contributed equally: Takahiro Yamane and Yoshikazu Kimura
†Correspondence concerning this article should be addressed to Mizuki Morita and Hiroshi Morimatsu. Email: mizuki@okayama-u.ac.jp, pb9b45wr@s.okayama-u.ac.jp
https://doi.org/10.1002/wjs.70472

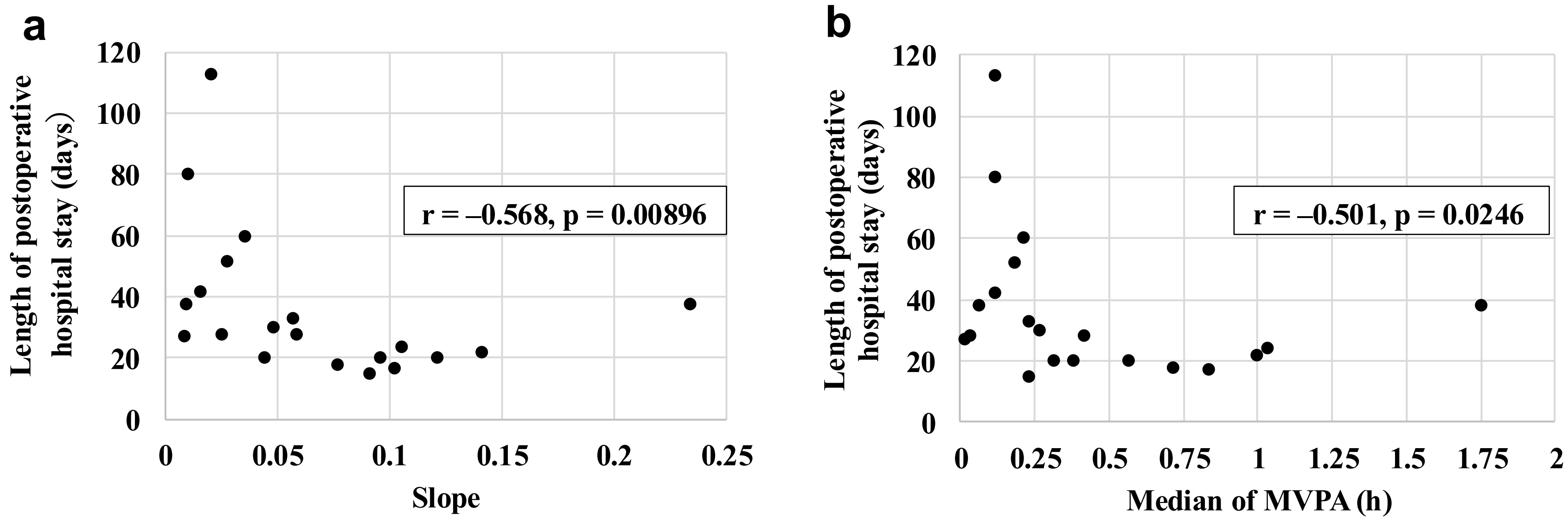

Figure S6 (a.) Association between slope of a straight regression line of changes in MVPA (calculated using MVPA data from postoperative day (POD) 1–13) and length of postoperative hospital stay. There is a statistically significant negative correlation between them shown in the box, (b.) Association between median of MVPA (calculated using MVPA data from POD 1–13) and length of postoperative hospital stay. There is a statistically significant negative correlation between them shown in the box.

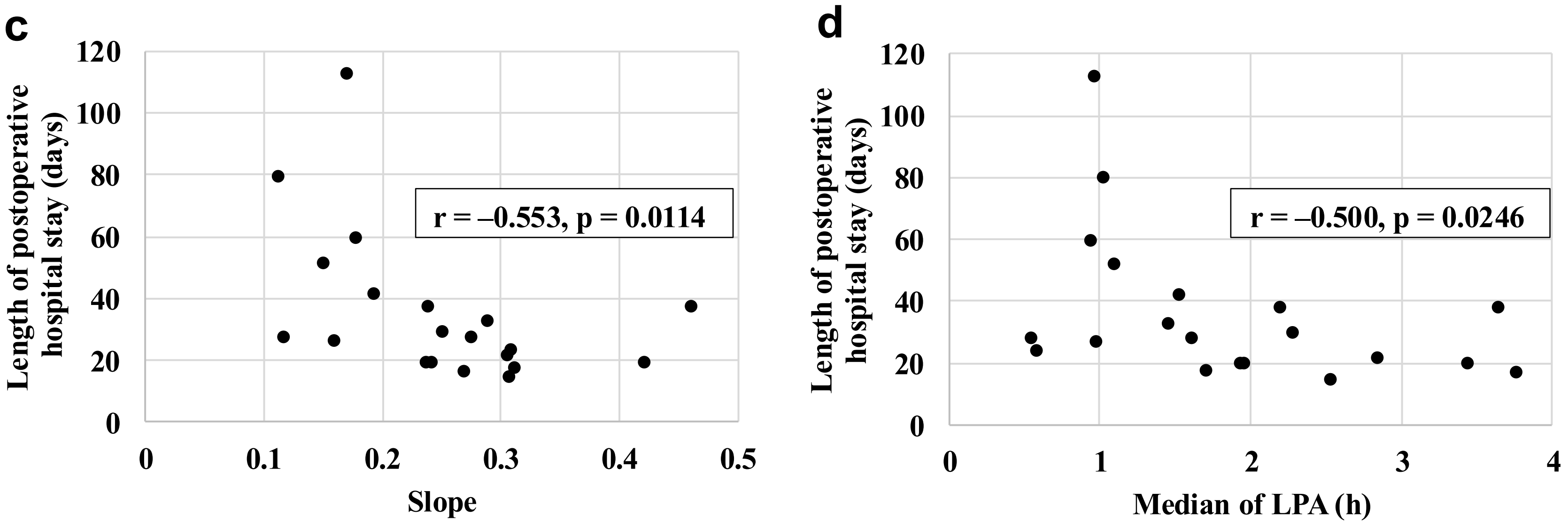

Figure S6 (c.) Association between slope of a straight regression line of changes in LPA (calculated using LPA data from POD 1–13) and length of postoperative hospital stay. There is a statistically significant negative correlation between them shown in the box, (d.) Association between median of LPA (calculated using LPA data from POD 1–13) and length of postoperative hospital stay. There is a statistically significant negative correlation between them shown in the box.

T. Yamane, Y. Kimura, M. Tetsutani, et al., "Indicators for Monitoring Recovery From Surgery to Discharge Using Accelerometer in Patients With Esophageal Cancer," *World Journal of Surgery* (2026): https://doi.org/10.1002/wjs.70472

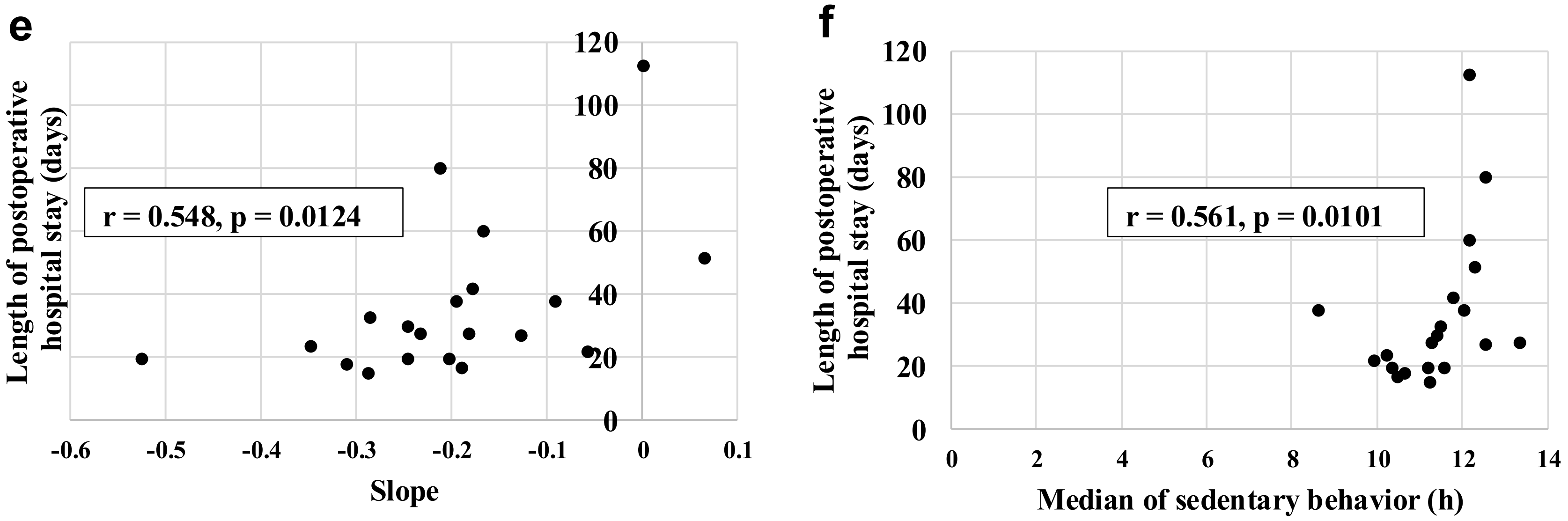

Figure S6 (e.) Association between slope of a straight regression line of changes in sedentary behavior (calculated using sedentary behavior data from POD 1–13) and length of postoperative hospital stay. There is a statistically significant positive correlation between them shown in the box, (f.) Association between median of sedentary behavior (calculated using sedentary behavior data from POD 1–13) and length of postoperative hospital stay. There is a statistically significant positive correlation between them shown in the box.

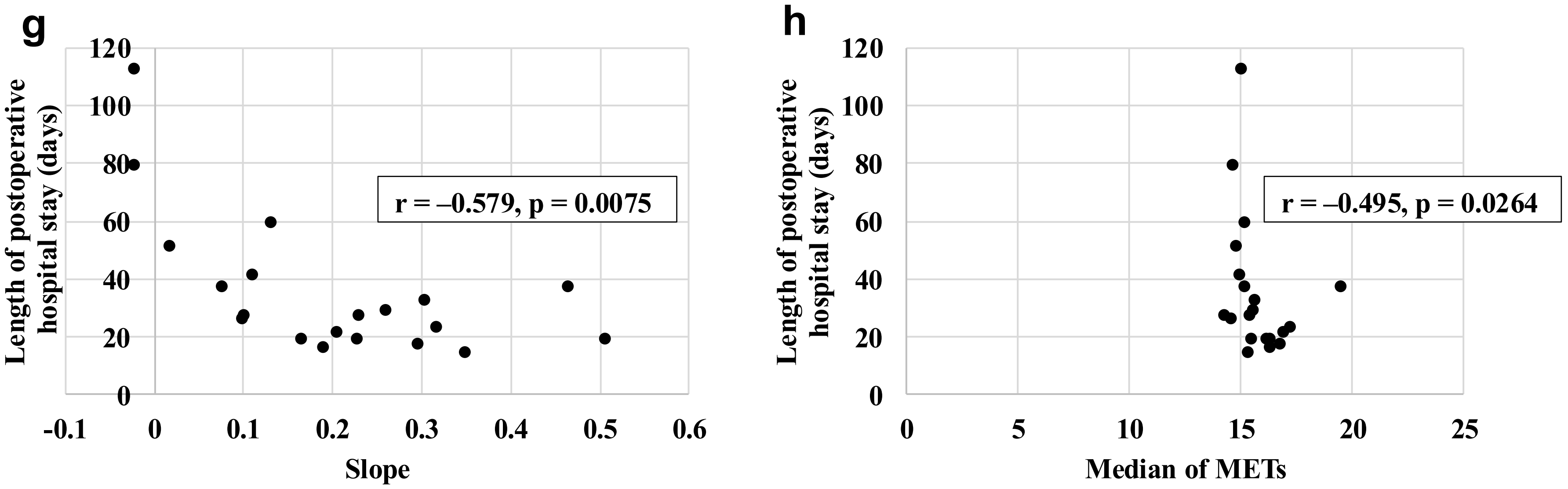

Figure S6 (g.) Association between slope of a straight regression line of changes in METs (calculated using METs data from POD 1–13) and length of postoperative hospital stay. There is a statistically significant negative correlation between them shown in the box, (h.) Association between median of METs (calculated using METs data from POD 1–13) and length of postoperative hospital stay. There is a statistically significant negative correlation between them shown in the box.

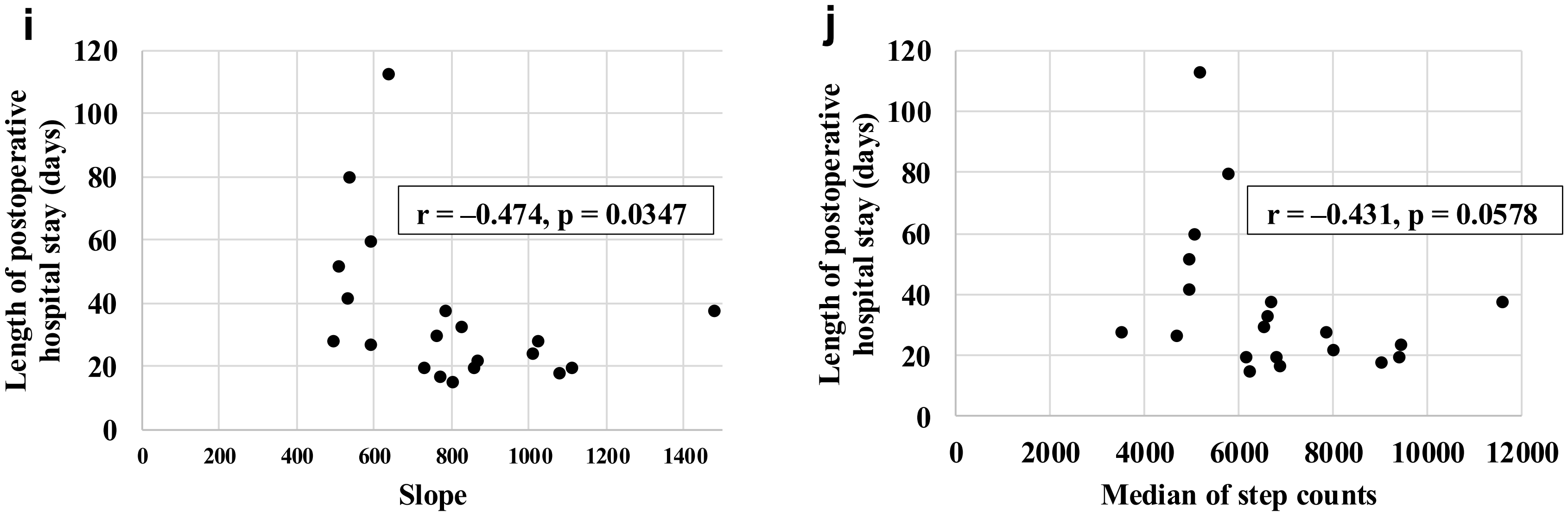

Figure S6 (i.) Association between slope of a straight regression line of changes in step counts (calculated using step counts data from POD 1–13) and length of postoperative hospital stay. There is a statistically significant negative correlation between them shown in the box, (j.) Association between median of step counts (calculated using step counts data from POD 1–13) and length of postoperative hospital stay.
