# Supplementary material for: Indicators for Monitoring Recovery From Surgery to Discharge Using Accelerometer in Patients With Esophageal Cancer
Source: World J Surg. 2026 Jul 2;50(8):2434–7. doi: 10.1002/wjs.70472 (PMC13460619; doi:10.1002/wjs.70472)
Supplement: Supplementary file 3 [file WJS-50-2434-s003.pdf]

Online Resource 6

Indicators for Monitoring Recovery From Surgery to Discharge Using Accelerometer in Patients With Esophageal Cancer

Takahiro Yamane[1]*, Yoshikazu Kimura[2]*, Manami Tetsutani[3], Shota Yamamura[3], Airu Ito[3], Moe Tanuma[3], Mina Honjoh[3], Yoshiko Moriwaki[2], Kazuhiro Noma[4], Shunsuke Tanabe[4], Naoaki Maeda[4], Yoshikazu Matsuoka[2], Mizuki Morita[1,3]†, Hiroshi Morimatsu[2]

[1] Department of Biomedical Informatics, Okayama University Graduate School of Interdisciplinary Science and Engineering in Health Systems
[2] Department of Anesthesiology and Resuscitology, Okayama University Graduate School of Medicine, Dentistry, and Pharmaceutical Sciences
[3] Faculty of Health Sciences, Okayama University Medical School
[4] Department of Gastroenterological Surgery, Okayama University Graduate School of Medicine, Dentistry and Pharmaceutical Sciences,
* These authors contributed equally: Takahiro Yamane and Yoshikazu Kimura
†Correspondence concerning this article should be addressed to Mizuki Morita and Hiroshi Morimatsu. Email: mizuki@okayama-u.ac.jp, pb9b45wr@s.okayama-u.ac.jp
https://doi.org/10.1002/wjs.70472

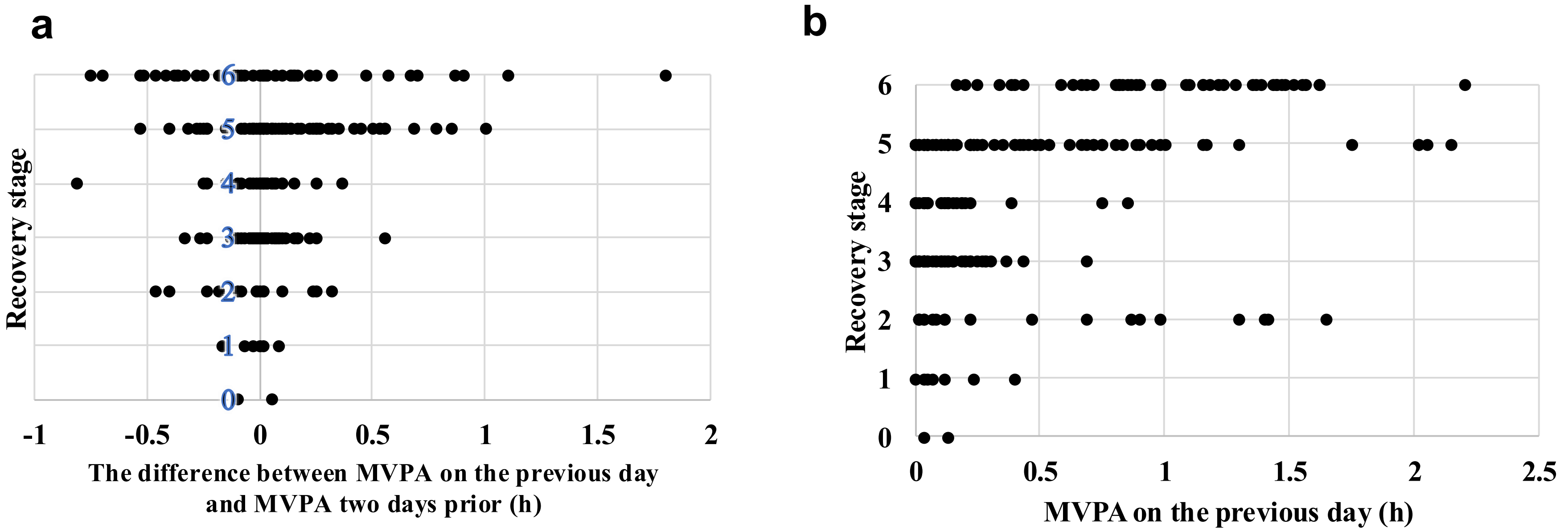

Figure S5 (a.) Relationship between the change in MVPA from two days prior to the previous day and recovery stage, (b.) Relationship between MVPA on the previous day and recovery stage.

c

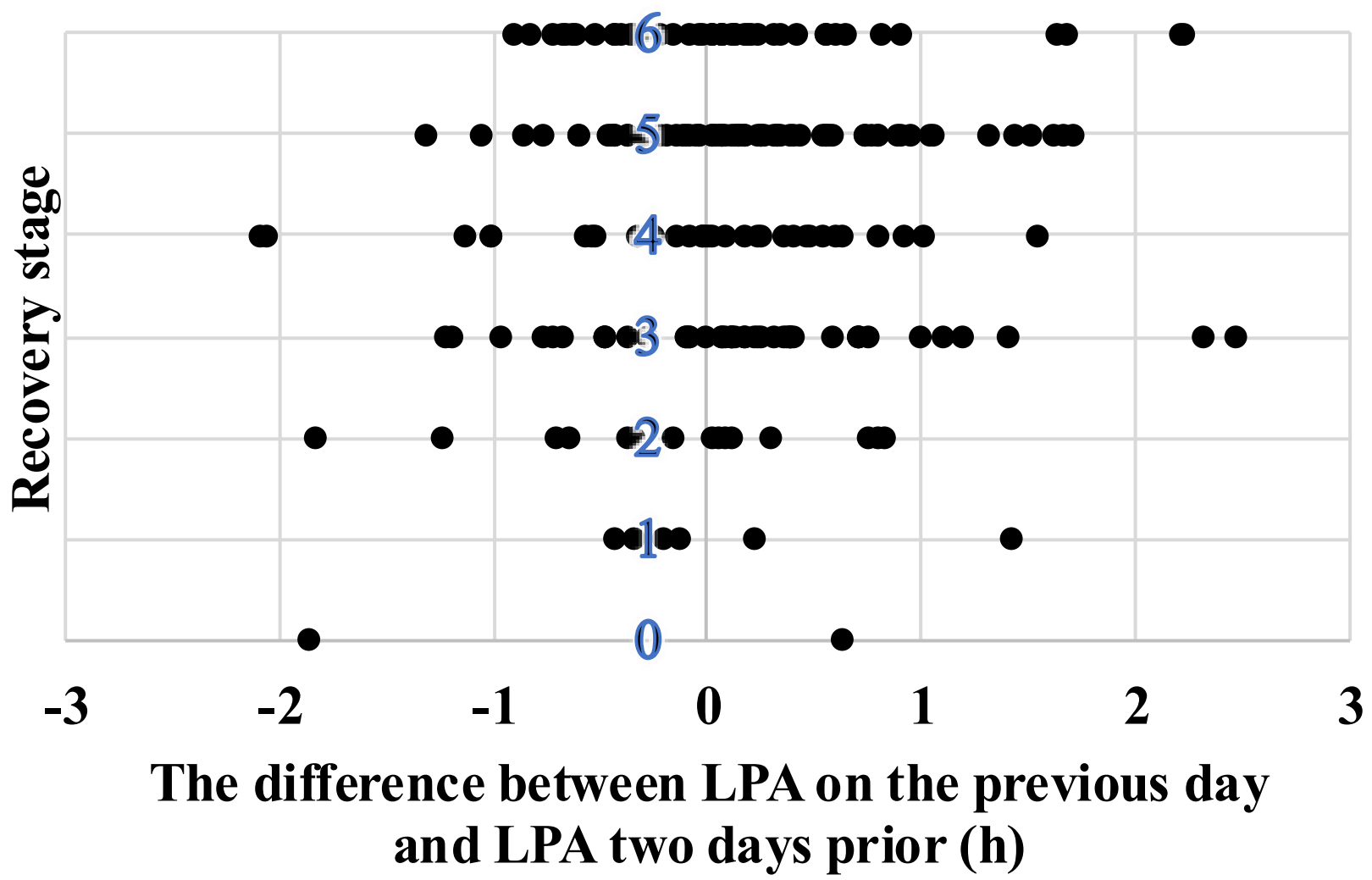

d

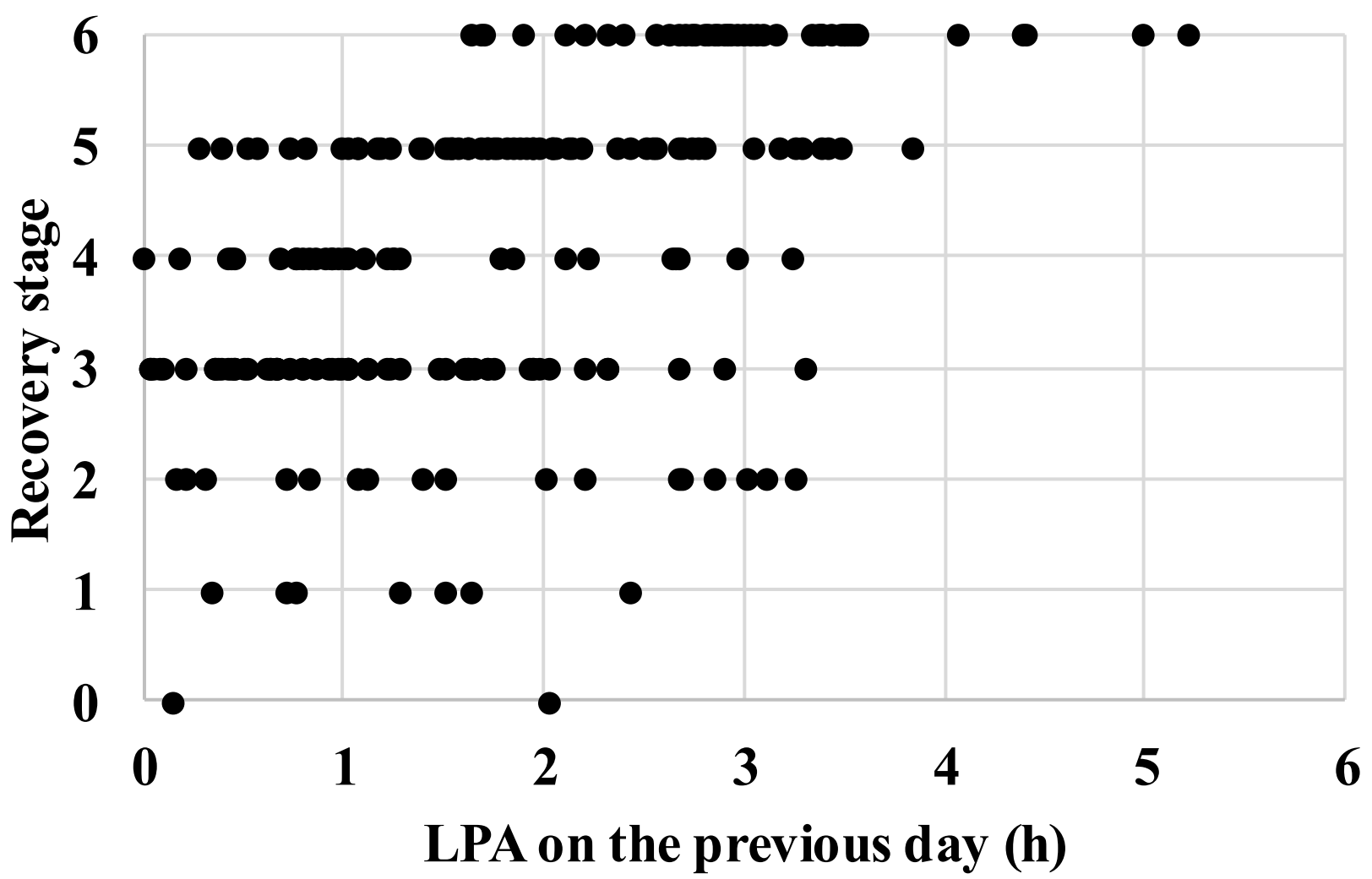

Figure S5 (c.) Relationship between the change in LPA from two days prior to the previous day and recovery stage, (d.) Relationship between LPA on the previous day and recovery stage.

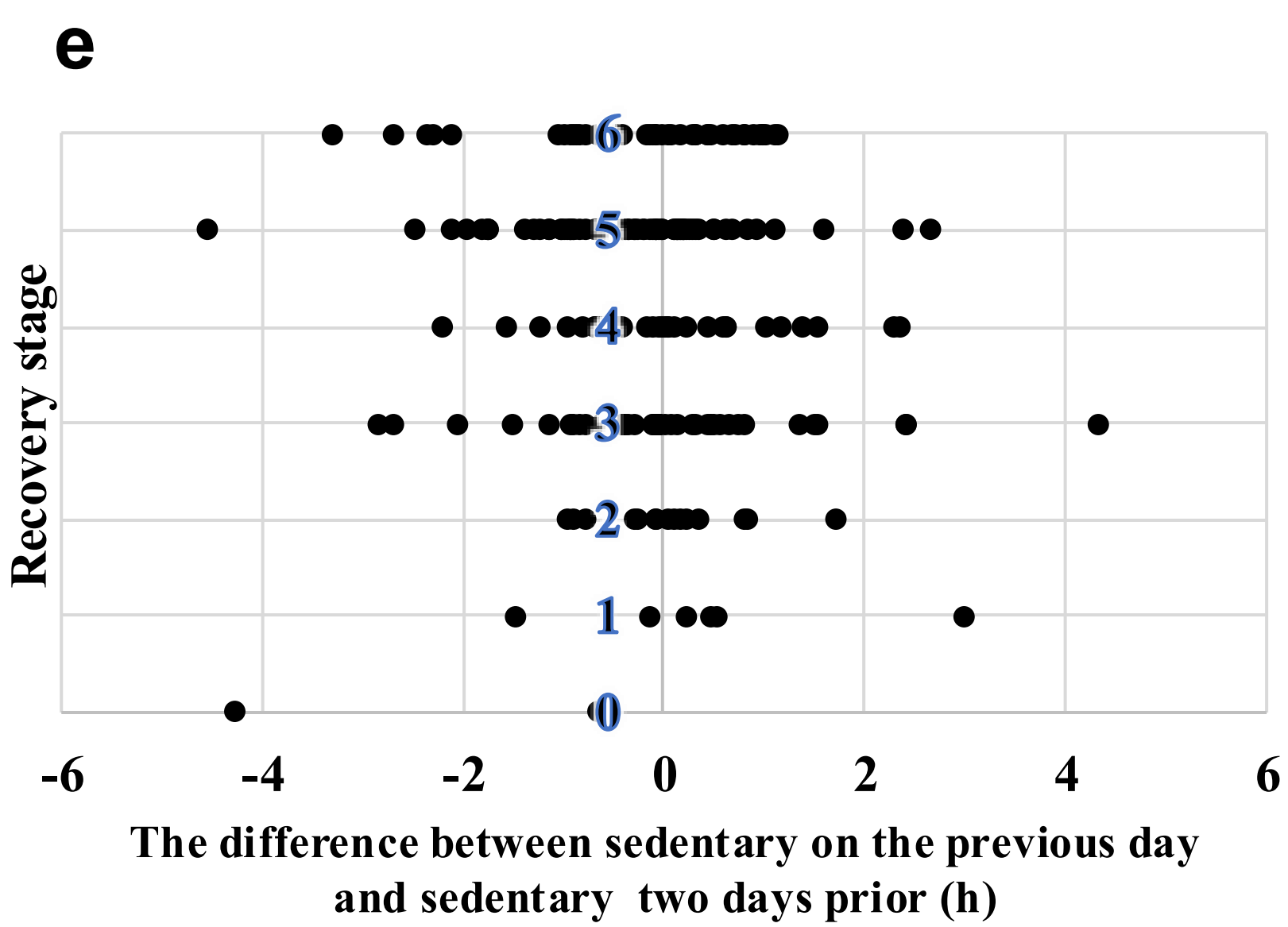

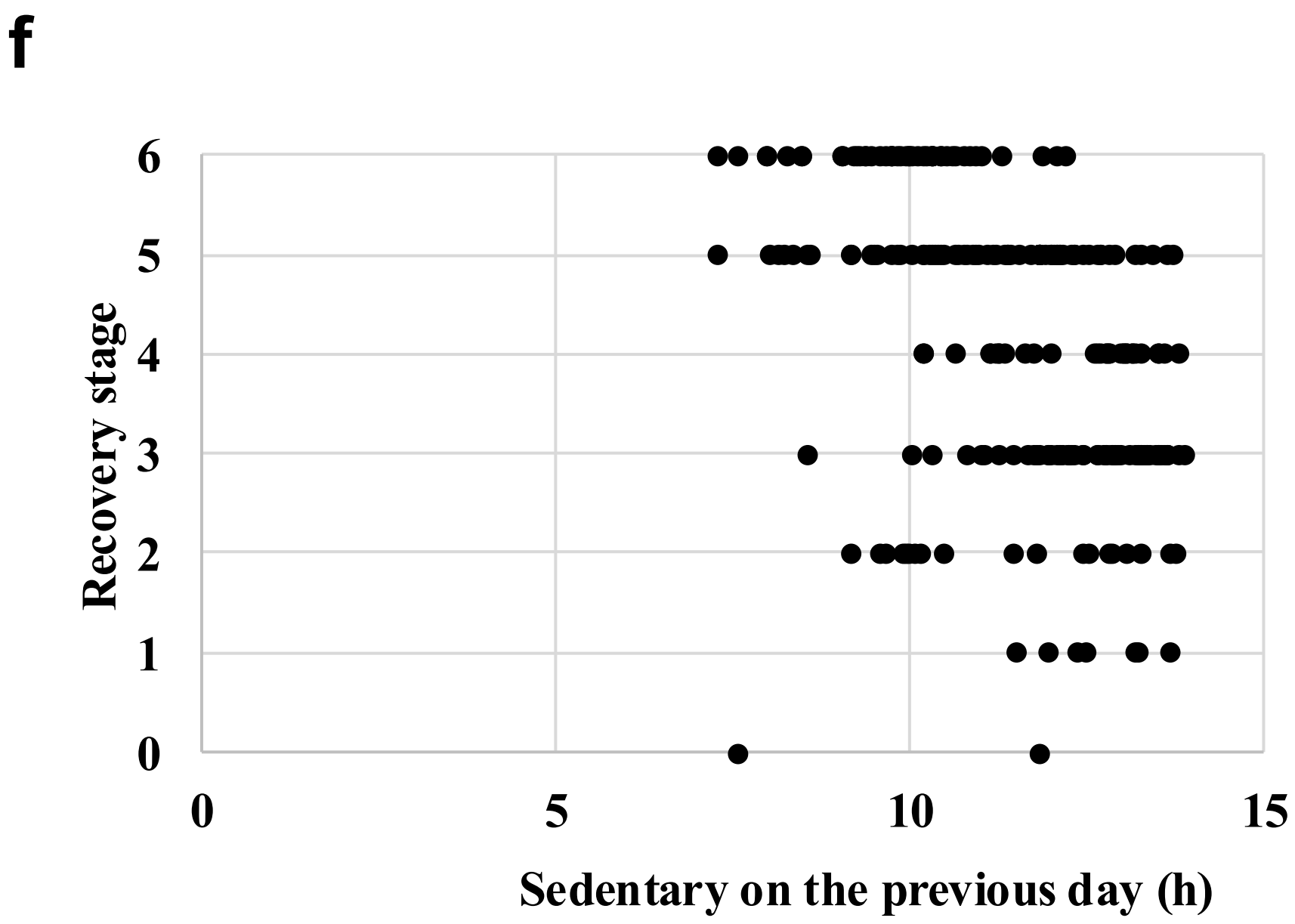

Figure S5 (e.) Relationship between the change in sedentary behavior from two days prior to the previous day and recovery stage, (f.) Relationship between sedentary behavior on the previous day and recovery stage.

T. Yamane, Y. Kimura, M. Tetsutani, et al., "Indicators for Monitoring Recovery From Surgery to Discharge Using Accelerometer in Patients With Esophageal Cancer," *World Journal of Surgery* (2026): https://doi.org/10.1002/wjs.70472

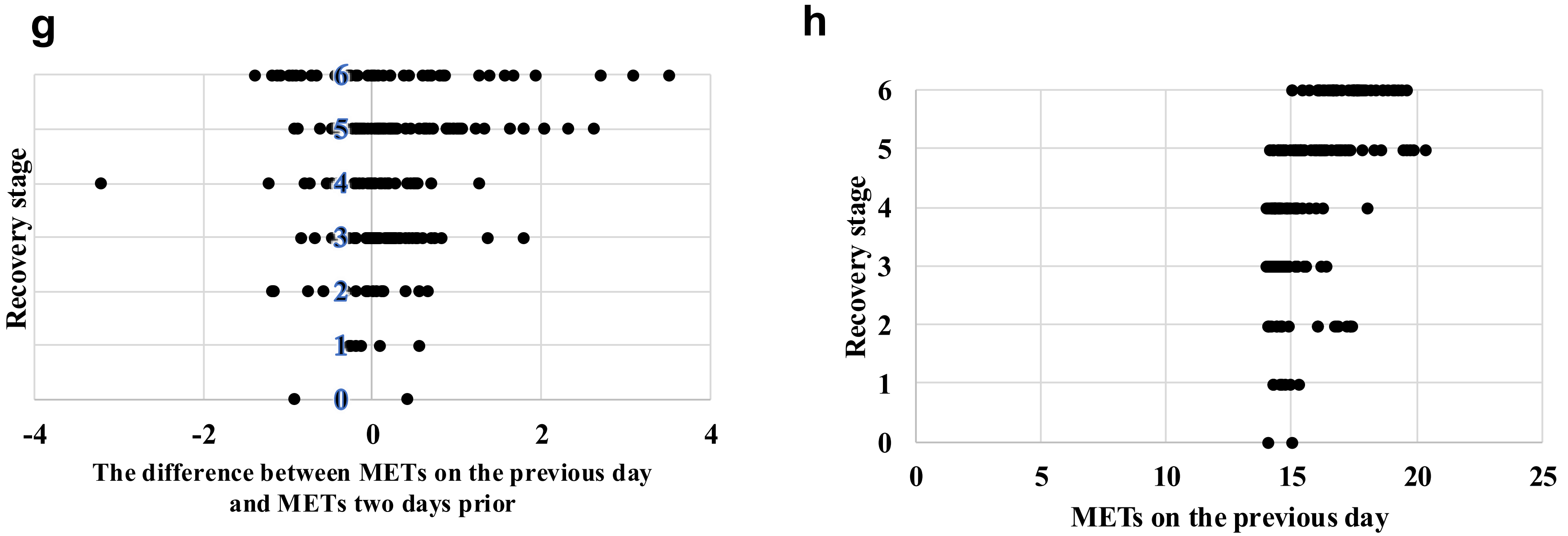

Figure S5 (g.) Relationship between the change in METs from two days prior to the previous day and recovery stage, (h.) Relationship between METs on the previous day and recovery stage.

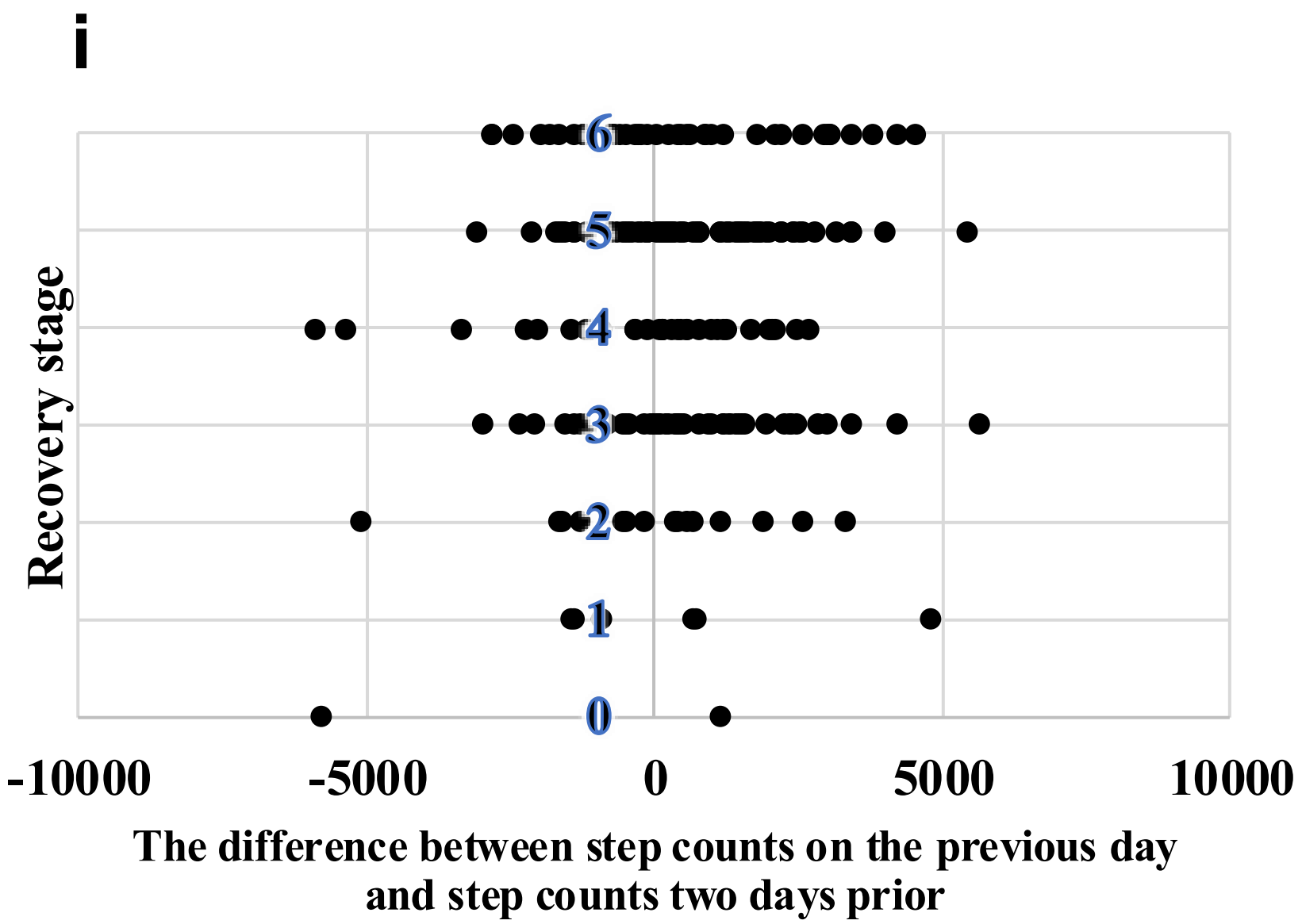

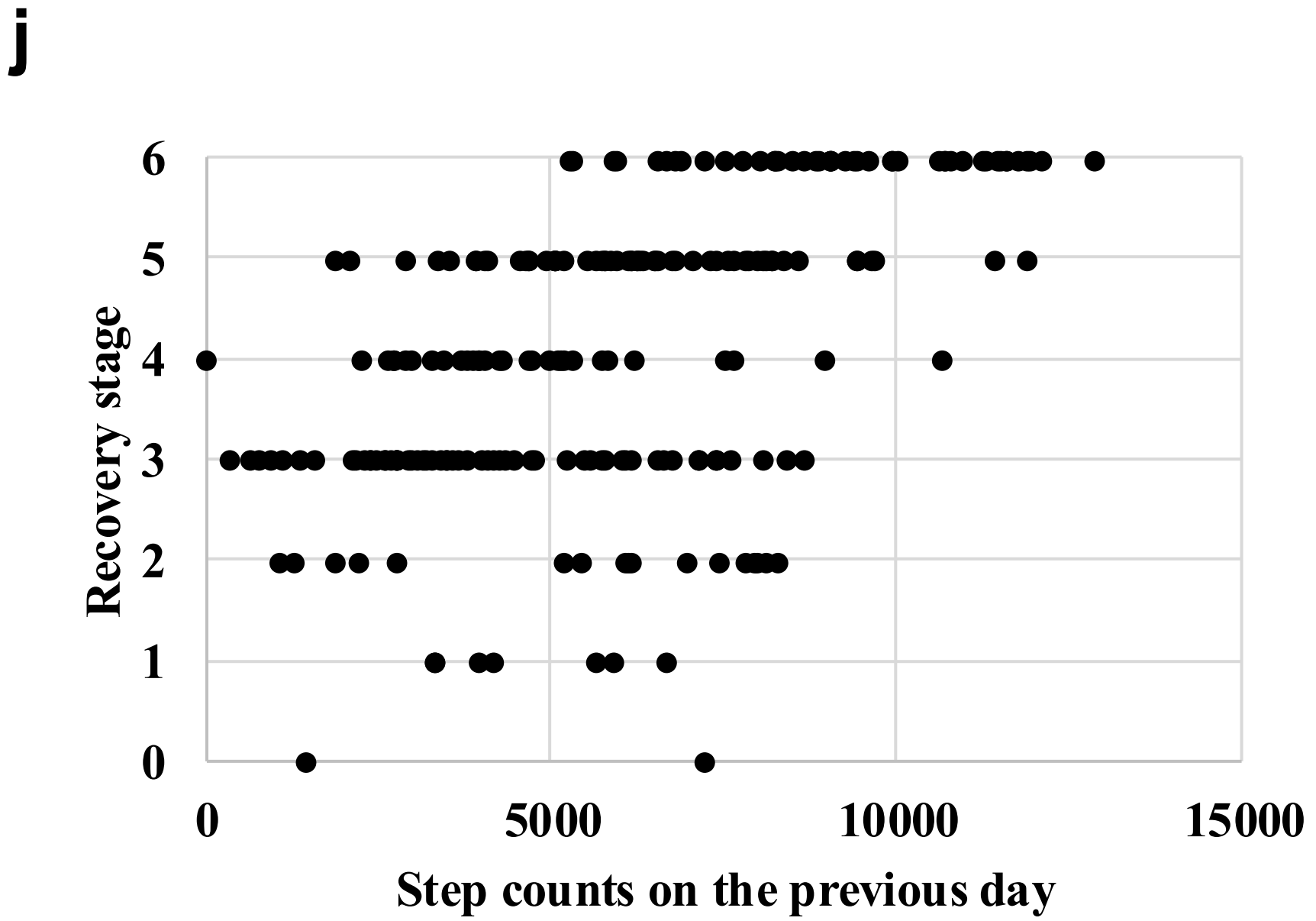

Figure S5 (i.) Relationship between the change in step counts from two days prior to the previous day and recovery stage, (j.) Relationship between step counts on the previous day and recovery stage.
