# Supplementary material for: Indicators for Monitoring Recovery From Surgery to Discharge Using Accelerometer in Patients With Esophageal Cancer
Source: World J Surg. 2026 Jul 2;50(8):2434–7. doi: 10.1002/wjs.70472 (PMC13460619; doi:10.1002/wjs.70472)
Supplement: Supplementary file 4 [file WJS-50-2434-s004.pdf]

T. Yamane, Y. Kimura, M. Tetsutani, et al., "Indicators for Monitoring Recovery From Surgery to Discharge Using Accelerometer in Patients With Esophageal Cancer," *World Journal of Surgery* (2026): https://doi.org/10.1002/wjs.70472

Results (Online Resource 3)

Participant characteristics and perioperative data are presented in Table S2.

TABLE S2 Patient characteristics

	n=20
Age, years (mean [SD])	72.9 (9.8)
Sex, n (%))	
Male	18 (90.0)
Female	2 (10.0)
BMI, kg/m^2 (mean [SD])	21.8 (3.0)
Physical status score, n (%)	
ASA I	1 (5.0)
ASA II	11 (55.0)
ASA III	8 (40.0)
Smoking (n (%))	
Yes	15 (75.0)
No	5 (25.0)
Stage (n (%))	
0	0 (0.0)
1	9 (45.0)
2	3 (15.0)
3	7 (35.0)
4	1 (5.0)
Intraoperative characteristics	
Operative time, h (median [IQR])	11.2 (1.87)
Blood loss, mL (median [IQR])	95 (127.5)
Postoperative characteristics	
Time to first ambulation, days (median [IQR])	3.0 (1.0)
Postoperative complications, n (%)	13 (65.0)
Postoperative delirium, n (%)	5 (25.0)
Postoperative LOS, days (median [IQR] days)	27.5 (19.0)

Abbreviations: SD, standard deviation; BMI, body mass index; ASA, American Society of Anesthesiologists; IQR, interquartile range; LOS, length of postoperative hospital stay.
