# Supplementary material for: Indicators for Monitoring Recovery From Surgery to Discharge Using Accelerometer in Patients With Esophageal Cancer
Source: World J Surg. 2026 Jul 2;50(8):2434–7. doi: 10.1002/wjs.70472 (PMC13460619; doi:10.1002/wjs.70472)
Supplement: Supplementary file 5 [file WJS-50-2434-s005.pdf]

T. Yamane, Y. Kimura, M. Tetsutani, et al., "Indicators for Monitoring Recovery From Surgery to Discharge Using Accelerometer in Patients With Esophageal Cancer," *World Journal of Surgery* (2026): https://doi.org/10.1002/wjs.70472

Results (Online Resource 5)

The categories and types of postoperative complications and number of patients in whom they occurred are presented in Table S3.

TABLE S3 Categories and types of postoperative complications and number of patients

Group of postoperative complications	Postoperative complications	Number of patients
Respiratory	Pleural effusion	4
	Pneumonia	2
	Tracheal compression due to gastric dilatation	1
	Organizing pneumonia	1
Circulatory	Atrial fibrillation	2
	Blood pressure drop	1
	Tachycardia	1
Digestive	Pyloric stenosis	3
	Gastric stasis	1
Surgical	Anastomotic leakage	2
	False aneurysm	1
Neurological	Recurrent laryngeal nerve palsy	1
