# Supplementary material for: Indicators for Monitoring Recovery From Surgery to Discharge Using Accelerometer in Patients With Esophageal Cancer
Source: World J Surg. 2026 Jul 2;50(8):2434–7. doi: 10.1002/wjs.70472 (PMC13460619; doi:10.1002/wjs.70472)
Supplement: Supplementary file 6 [file WJS-50-2434-s006.pdf]

Methods (Online Resource 2)

Trajectory of recovery and physical activity

A recovery trajectory was created for each patient using the rehabilitation records. First, each item in the rehabilitation records was classified into recovery stages ranging from 0 to 6. Ambulating to the rehabilitation room was considered the most advanced stage of recovery and was classified as 6 (Table S1). These stages were later found to correspond closely to a simplified version of the ICU Mobility Scale (IMS), an 11-point scale used in the intensive care unit (ICU) [1]. Next, the postoperative days were plotted on the horizontal axis and the recovery stages were plotted on the vertical axis to create trajectories (Figure S1).

Patients whose trajectory monotonically increased and reached recovery stage 6 during the period from postoperative day (POD) 1 to POD13 were classified as the "smooth recovery" group. Here, "monotonic increase" was defined as the rehabilitation stage remaining the same or progressing over time after surgery. However, a single instance of a one-stage decline in recovery stage was permitted. In contrast, patients who did not reach recovery stage 6 during the period from POD1 to POD 13 were classified as the "non-smooth recovery" group.

Additionally, one patient who reached recovery stage 6 by POD13 but exhibited a decline that did not meet the criterion of "only one instance of a one-stage decline" was classified as belonging to the "neither group." This patient exhibited two instances of decline, each involving a decrease of two or more recovery stages. Specifically, the patient developed atrial fibrillation on POD 6, accompanied by exacerbated pain, resulting in a decline in recovery stage from stage 5 (toilet ambulation) on POD 5 to stage 2 (bed-based rehabilitation) on POD 6. On POD 7, pain improved following regular administration of intravenous acetaminophen, and the patient was able to ambulate twice around the ward, corresponding to recovery stage 4. However, on POD 8, due to general discomfort associated with poor sleep on the previous night, rehabilitation was limited to bed-based

activity, and the recovery stage declined again to stage 2. Rehabilitation remained bed-based until POD 12; however, on POD 13, the patient was able to attend the rehabilitation room and participate in rehabilitation, reaching recovery stage 6.

TABLE S1 Rehabilitation records and recovery stages

Recovery stage	Records
0	Reintubation for mechanical ventilation
1	Decannulation<br>Cardiac sitting position
2	Edge-of-bed sitting<br>Rehabilitation on the bed
3	Ambulation in standing position<br>Ambulation in intensive care unit (ICU)<br>Ambulation with a walker<br>Ambulation with IV stand<br>Standing march
4	Ambulation in the hospital
5	Toilet ambulation
6	Ambulation to the rehabilitation room

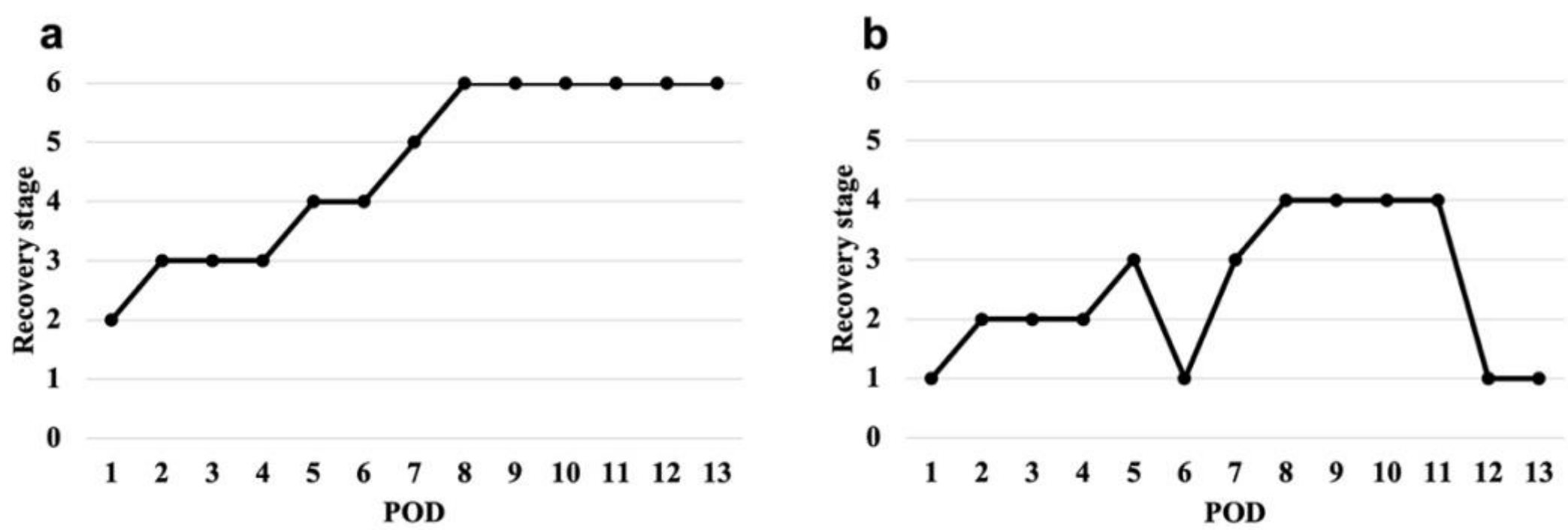

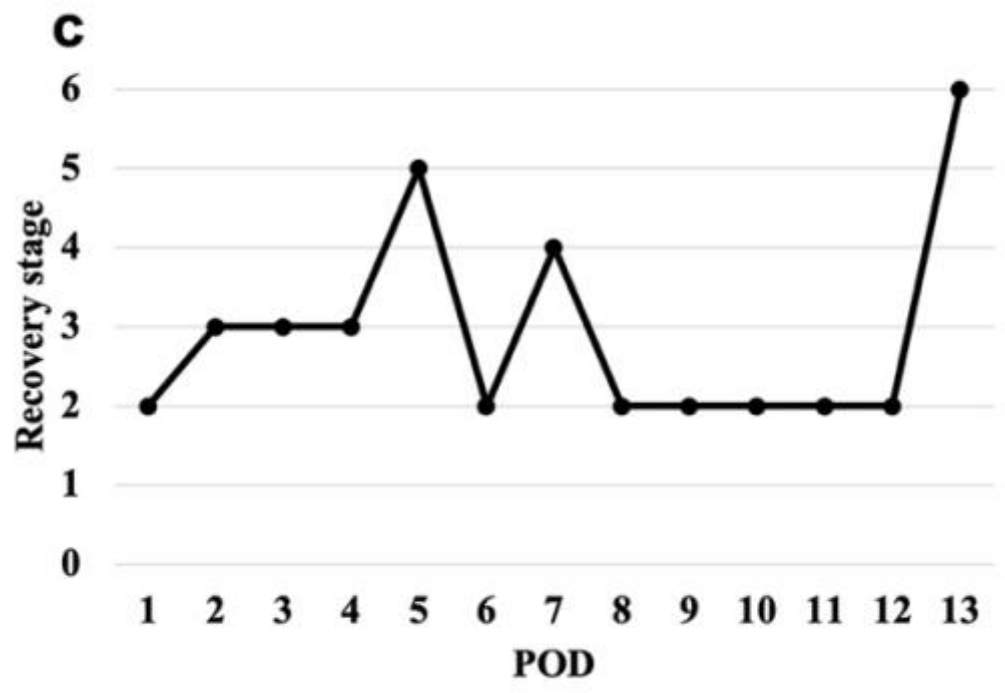

FIGURE S1 Examples of patterns of changes in recovery stage from postoperative day (POD) 1 to POD 13: (a.) smooth recovery group, (b.) non-smooth recovery group, (c.) neither group

A trajectory of MVPA for each patient was created, plotting the postoperative days from POD 1 to POD 13 on the horizontal axis and MVPA on the vertical axis (Figure S2). A straight regression line was made from the trajectory (Figure S3). As it was assumed that the patient would not move immediately after surgery, the line was adjusted to pass through the point of origin (0, 0). Trajectories and straight regression lines of LPA, sedentary behavior, METs, and step counts were also created in the same manner, except that the regression lines were not adjusted for sedentary behavior and METs, as these two indices could be counted even on the day of surgery.

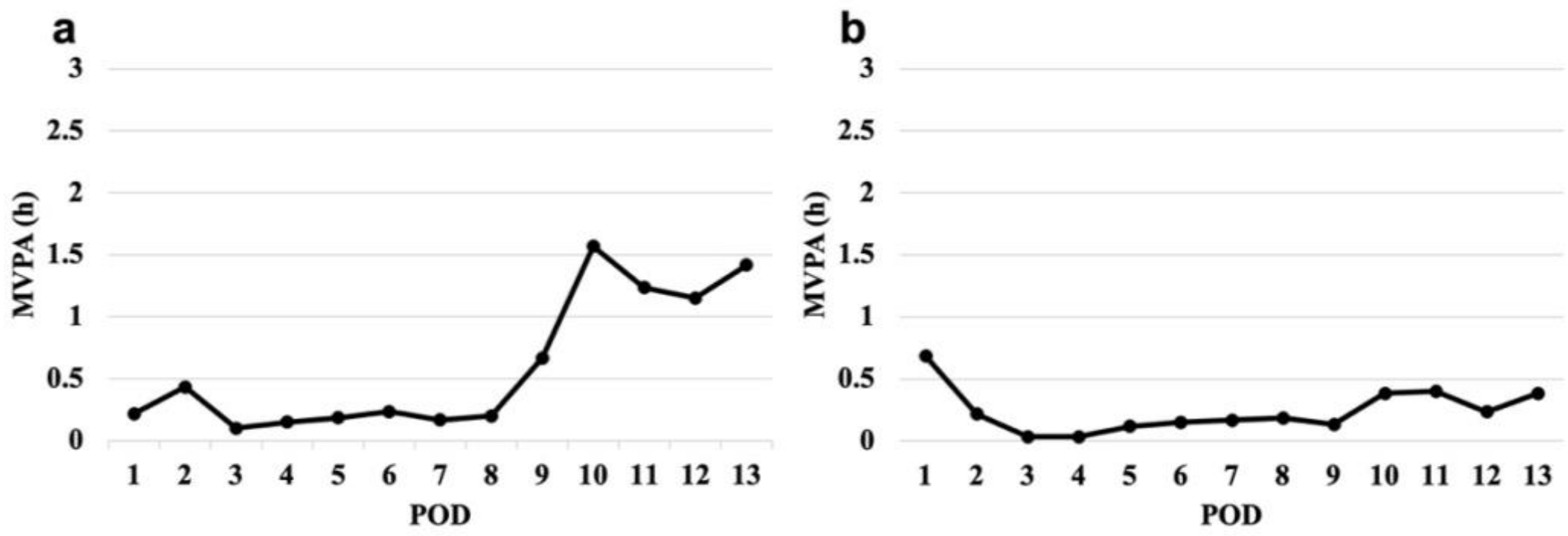

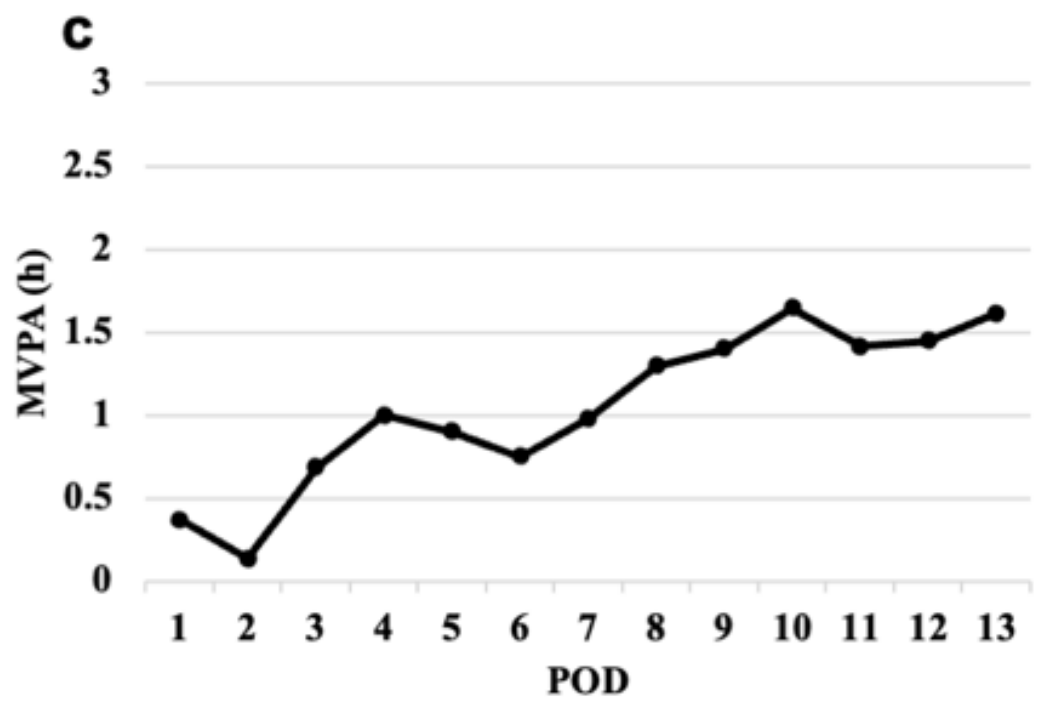

FIGURE S2 Examples of patterns of changes in MVPA from POD 1 to POD 13: (a.) smooth recovery group, (b.) non-smooth recovery group, (c.) neither group

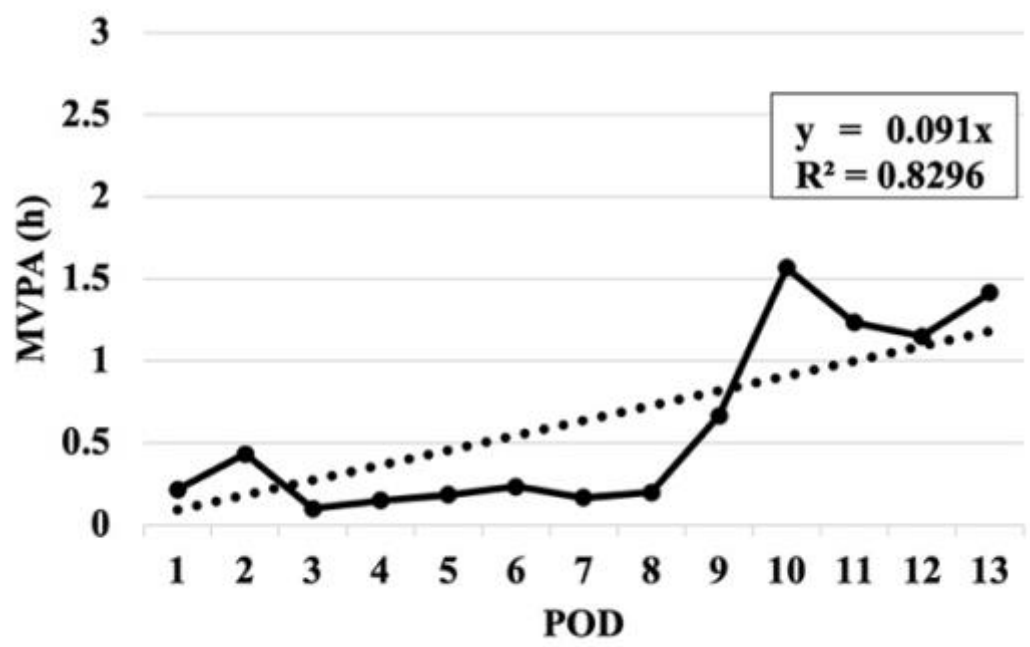

FIGURE S3 Example of a straight regression line of changes in MVPA. The straight regression line was adjusted so that it would pass through the point of origin (0, 0).

References

1. Hodgson C, Needham D, Haines K, et al. (2014) Feasibility and inter-rater reliability of the ICU Mobility Scale. *Heart & Lung* 43(1):19–24. https://doi.org/10.1016/j.hrtlng.2013.11.003.
