# Supplementary material for: Indicators for Monitoring Recovery From Surgery to Discharge Using Accelerometer in Patients With Esophageal Cancer
Source: World J Surg. 2026 Jul 2;50(8):2434–7. doi: 10.1002/wjs.70472 (PMC13460619; doi:10.1002/wjs.70472)

Online Resource 4

Indicators for Monitoring Recovery From Surgery to Discharge Using Accelerometer in Patients With Esophageal Cancer

Takahiro Yamane[1]*, Yoshikazu Kimura[2]*, Manami Tetsutani[3], Shota Yamamura[3], Airu Ito[3], Moe Tanuma[3], Mina Honjoh[3], Yoshiko Moriwaki[2], Kazuhiro Noma[4], Shunsuke Tanabe[4], Naoaki Maeda[4], Yoshikazu Matsuoka[2], Mizuki Morita[1,3]†, Hiroshi Morimatsu[2]

[1] Department of Biomedical Informatics, Okayama University Graduate School of Interdisciplinary Science and Engineering in Health Systems
[2] Department of Anesthesiology and Resuscitology, Okayama University Graduate School of Medicine, Dentistry, and Pharmaceutical Sciences
[3] Faculty of Health Sciences, Okayama University Medical School
[4] Department of Gastroenterological Surgery, Okayama University Graduate School of Medicine, Dentistry and Pharmaceutical Sciences,
* These authors contributed equally: Takahiro Yamane and Yoshikazu Kimura
†Correspondence concerning this article should be addressed to Mizuki Morita and Hiroshi Morimatsu. Email: mizuki@okayama-u.ac.jp, pb9b45wr@s.okayama-u.ac.jp
https://doi.org/10.1002/wjs.70472

Figure S4 Patterns of changes from postoperative day (POD) 1 to POD 13 of each participant in (a.) recovery stage, (b.) moderate-to-vigorous intensity physical activity (MVPA), (c.) light-intensity physical activity (LPA), (d.) sedentary, (e.) metabolic equivalents of task (METs), (f.) steps counts. AC-xx in the upper left corner indicates the case number. Next to the case number is the name of the groups classified based on the rehabilitation records.

AC-01: smooth recovery

T. Yamane, Y. Kimura, M. Tetsutani, et al., "Indicators for Monitoring Recovery From Surgery to Discharge Using Accelerometer in Patients With Esophageal Cancer," *World Journal of Surgery* (2026): https://doi.org/10.1002/wjs.70472

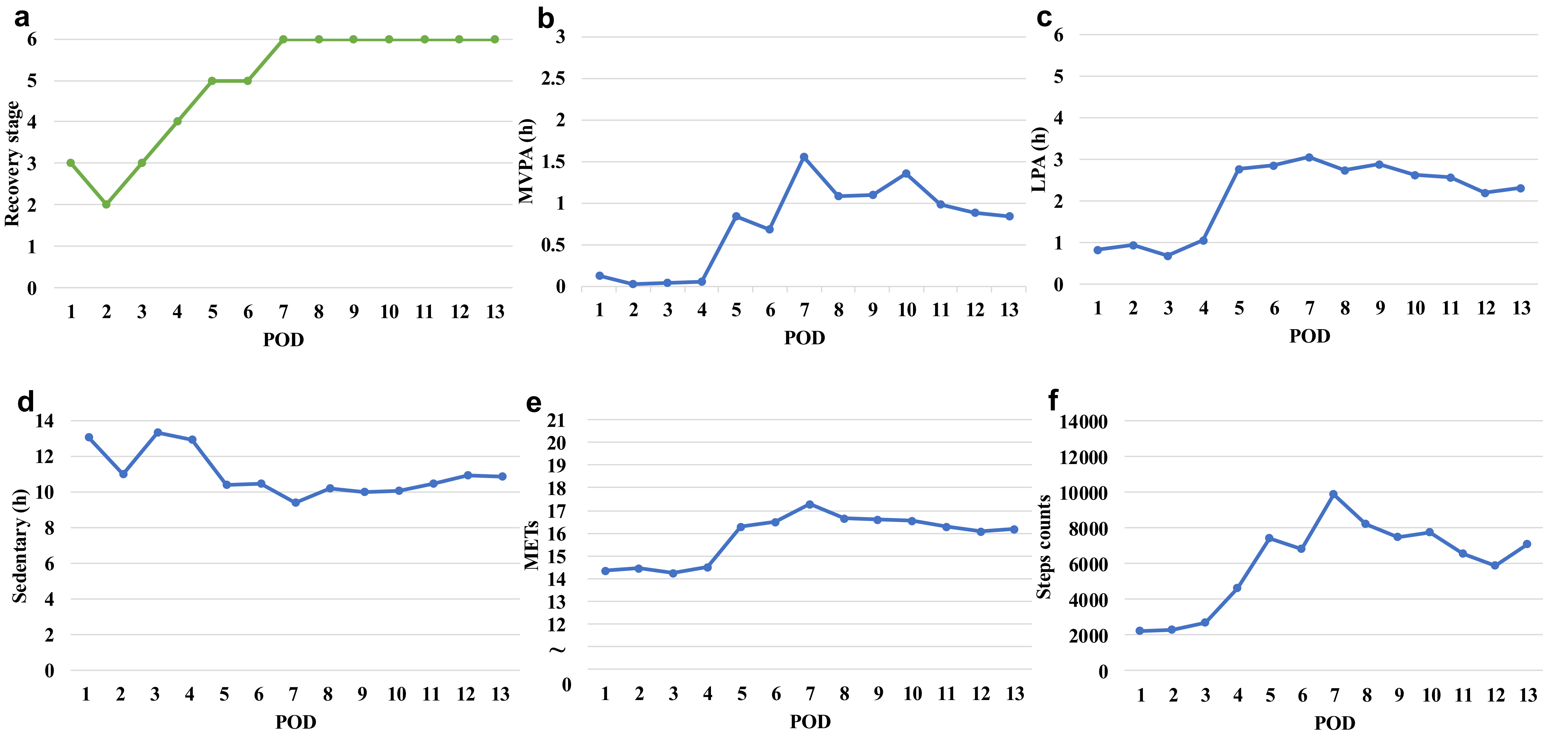

AC-02: neither group

T. Yamane, Y. Kimura, M. Tetsutani, et al., "Indicators for Monitoring Recovery From Surgery to Discharge Using Accelerometer in Patients With Esophageal Cancer," *World Journal of Surgery* (2026): https://doi.org/10.1002/wjs.70472

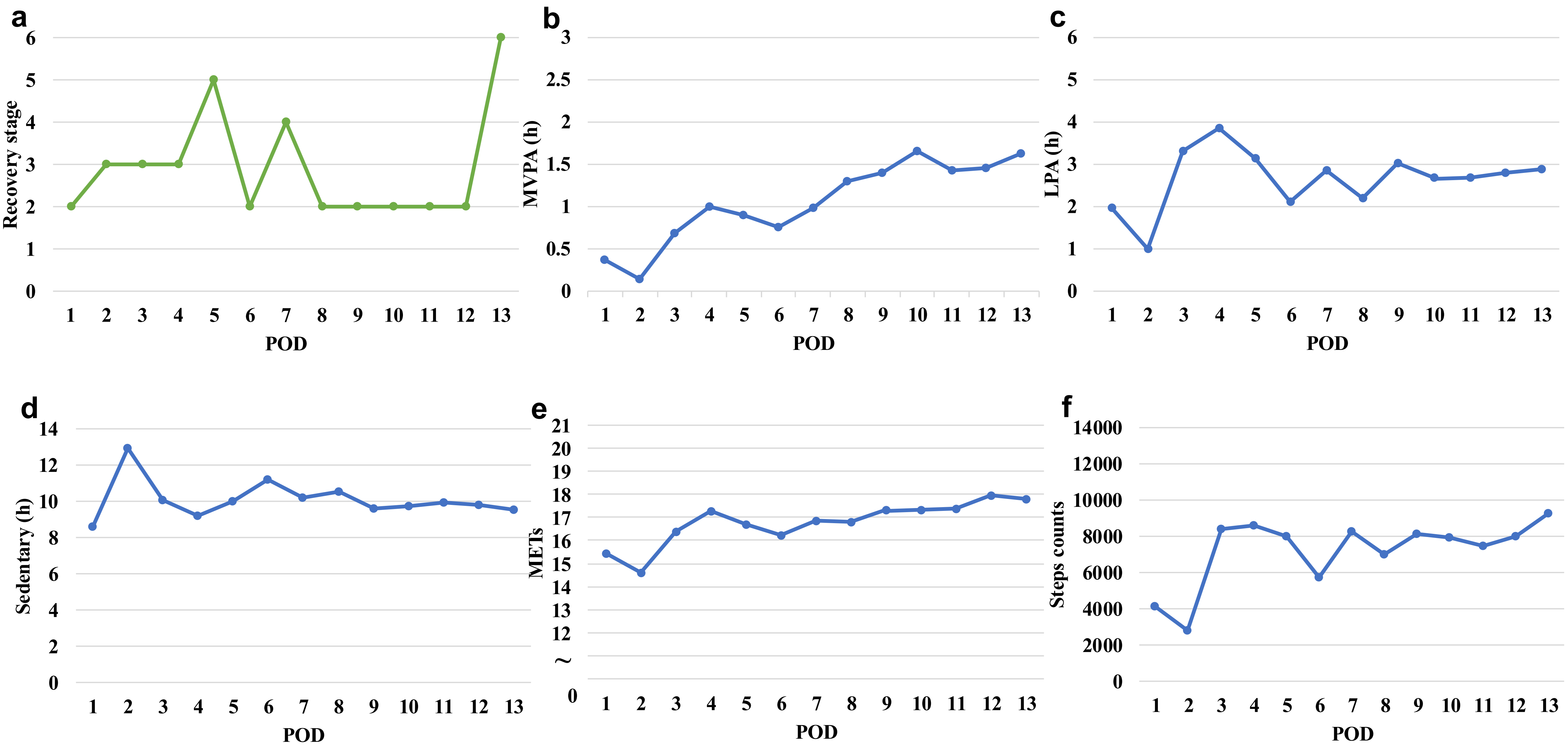

AC-03: non-smooth recovery

T. Yamane, Y. Kimura, M. Tetsutani, et al., "Indicators for Monitoring Recovery From Surgery to Discharge Using Accelerometer in Patients With Esophageal Cancer," *World Journal of Surgery* (2026): https://doi.org/10.1002/wjs.70472

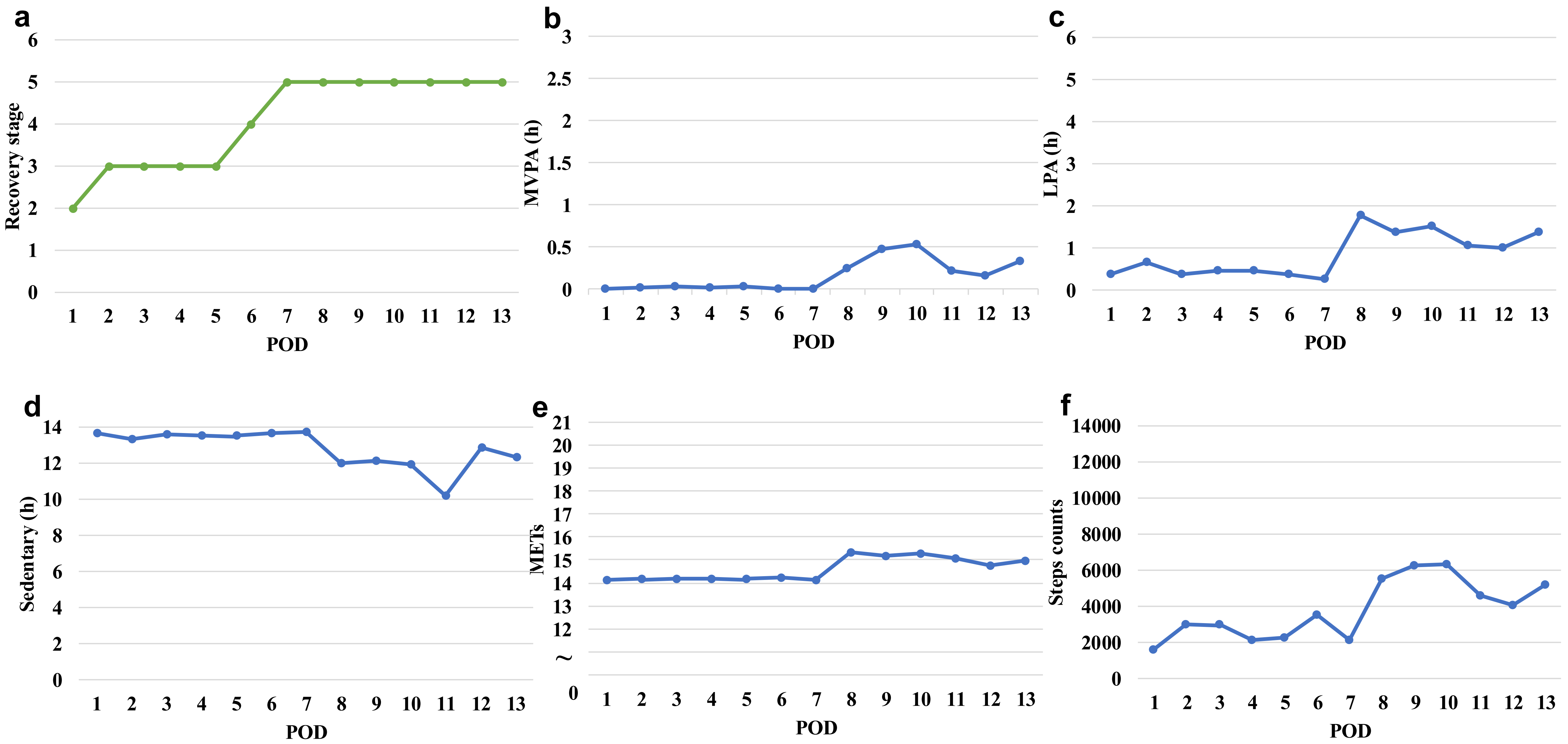

AC-04: non-smooth recovery

T. Yamane, Y. Kimura, M. Tetsutani, et al., "Indicators for Monitoring Recovery From Surgery to Discharge Using Accelerometer in Patients With Esophageal Cancer," *World Journal of Surgery* (2026): https://doi.org/10.1002/wjs.70472

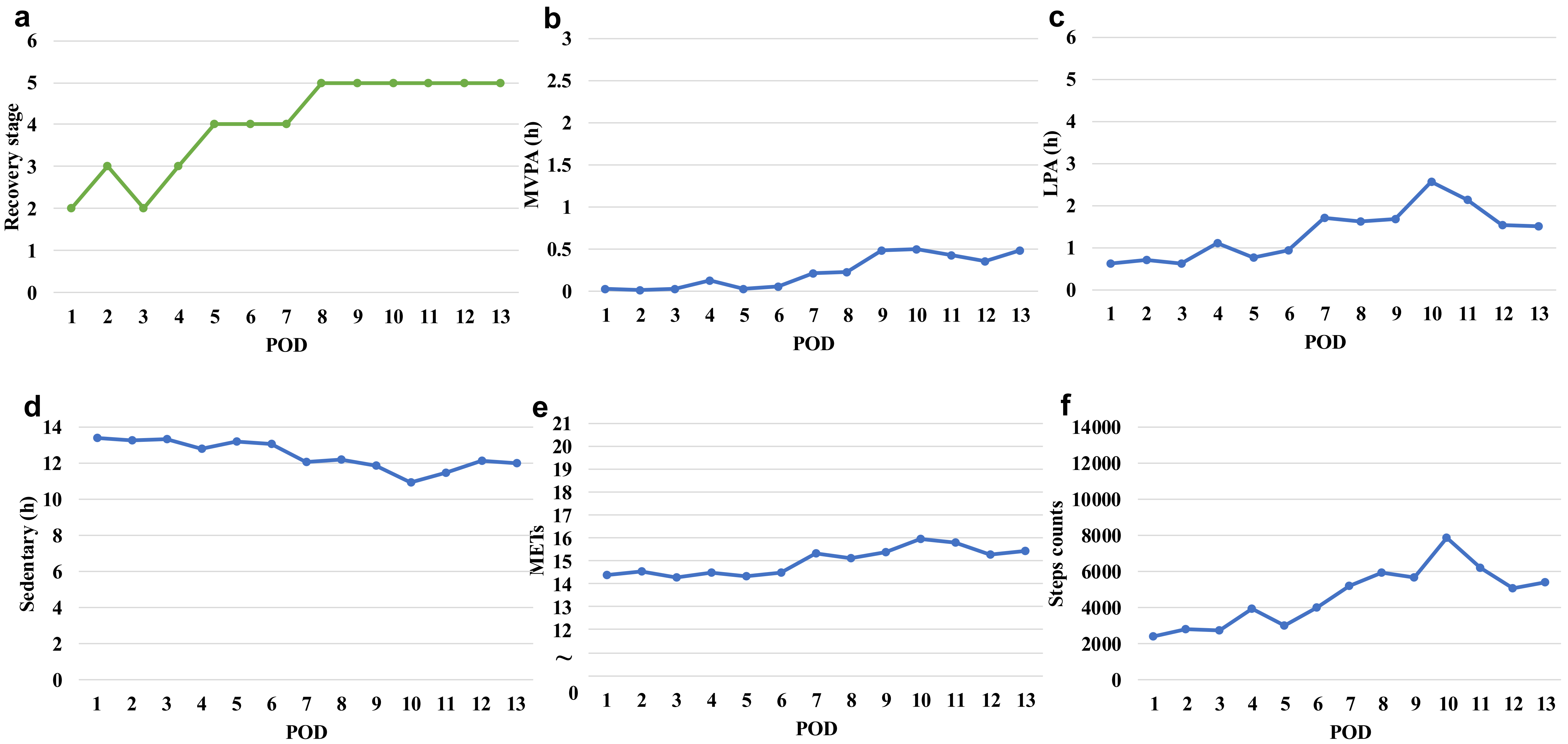

AC-05: non-smooth recovery

T. Yamane, Y. Kimura, M. Tetsutani, et al., “Indicators for Monitoring Recovery From Surgery to Discharge Using Accelerometer in Patients With Esophageal Cancer,” *World Journal of Surgery* (2026): https://doi.org/10.1002/wjs.70472

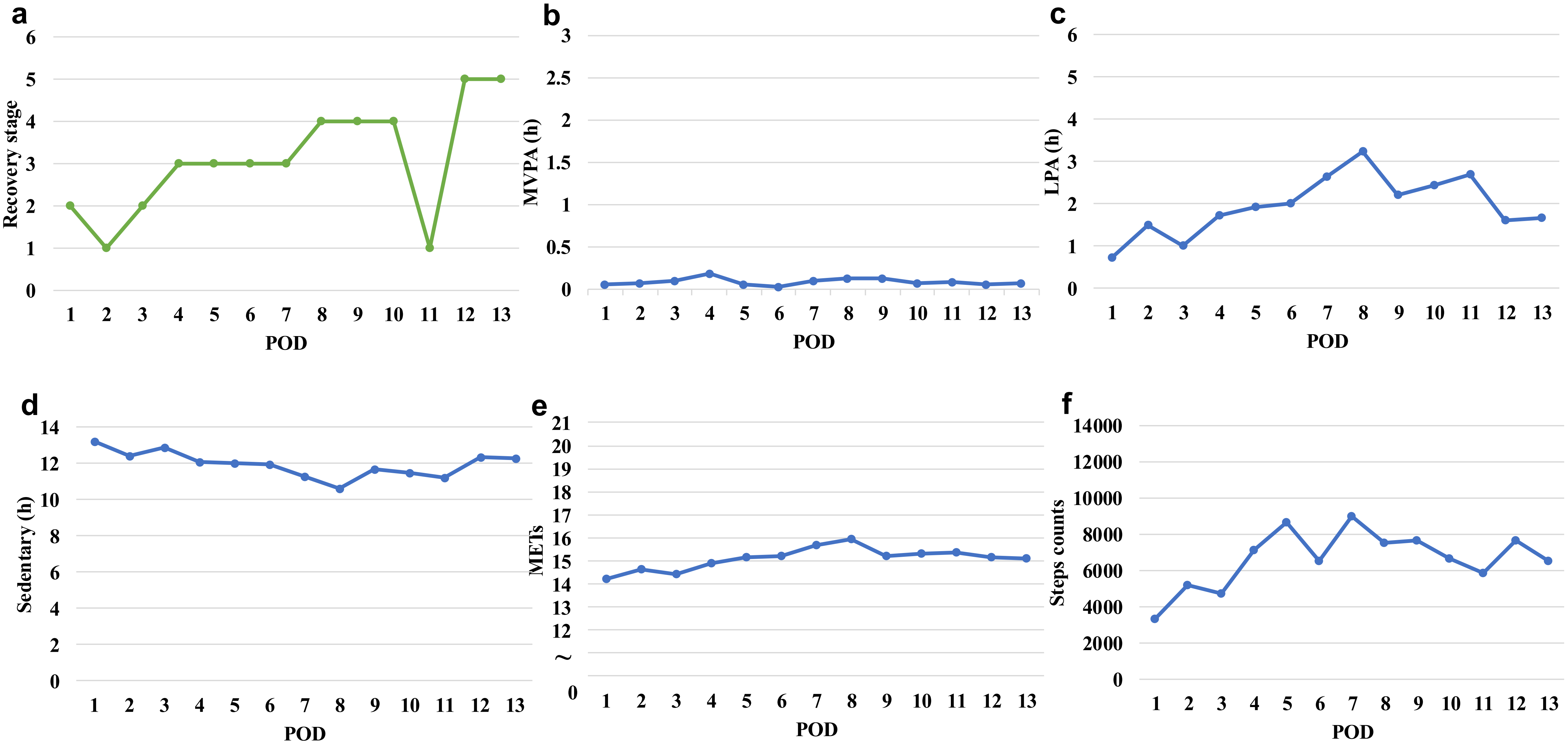

AC-06: smooth recovery

T. Yamane, Y. Kimura, M. Tetsutani, et al., "Indicators for Monitoring Recovery From Surgery to Discharge Using Accelerometer in Patients With Esophageal Cancer," *World Journal of Surgery* (2026): https://doi.org/10.1002/wjs.70472

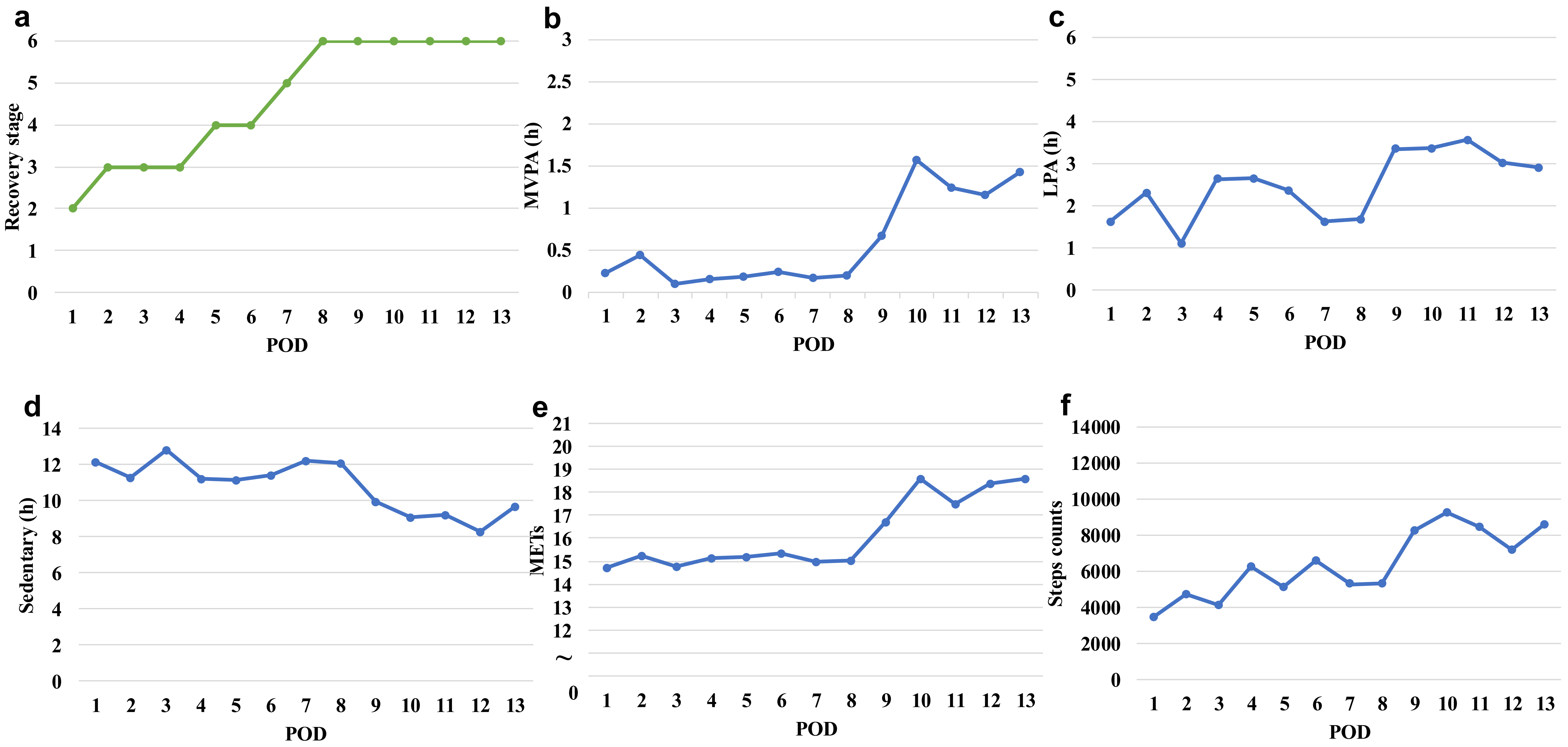

AC-07: non-smooth recovery

T. Yamane, Y. Kimura, M. Tetsutani, et al., "Indicators for Monitoring Recovery From Surgery to Discharge Using Accelerometer in Patients With Esophageal Cancer," *World Journal of Surgery* (2026): https://doi.org/10.1002/wjs.70472

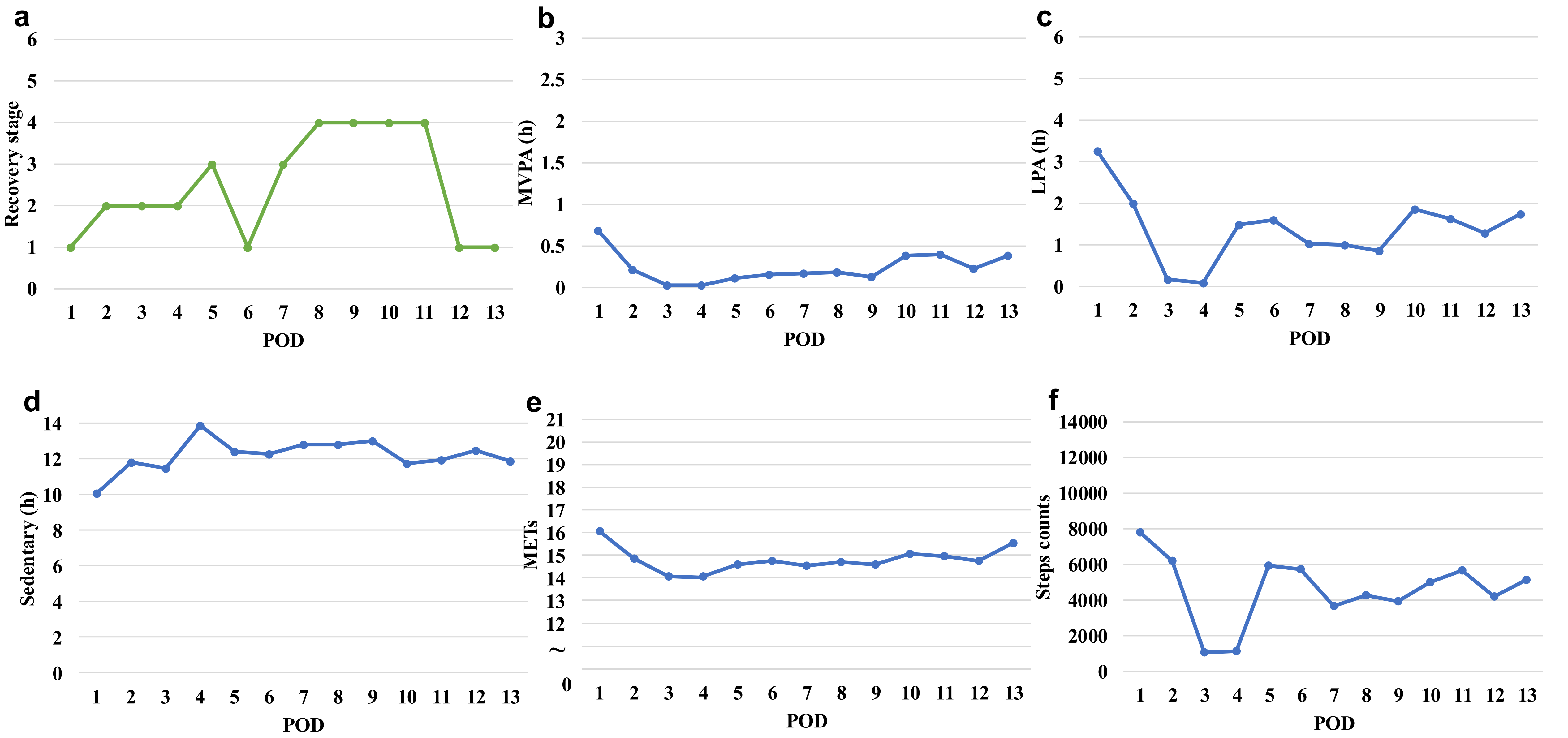

AC-08: smooth recovery

T. Yamane, Y. Kimura, M. Tetsutani, et al., "Indicators for Monitoring Recovery From Surgery to Discharge Using Accelerometer in Patients With Esophageal Cancer," *World Journal of Surgery* (2026): https://doi.org/10.1002/wjs.70472

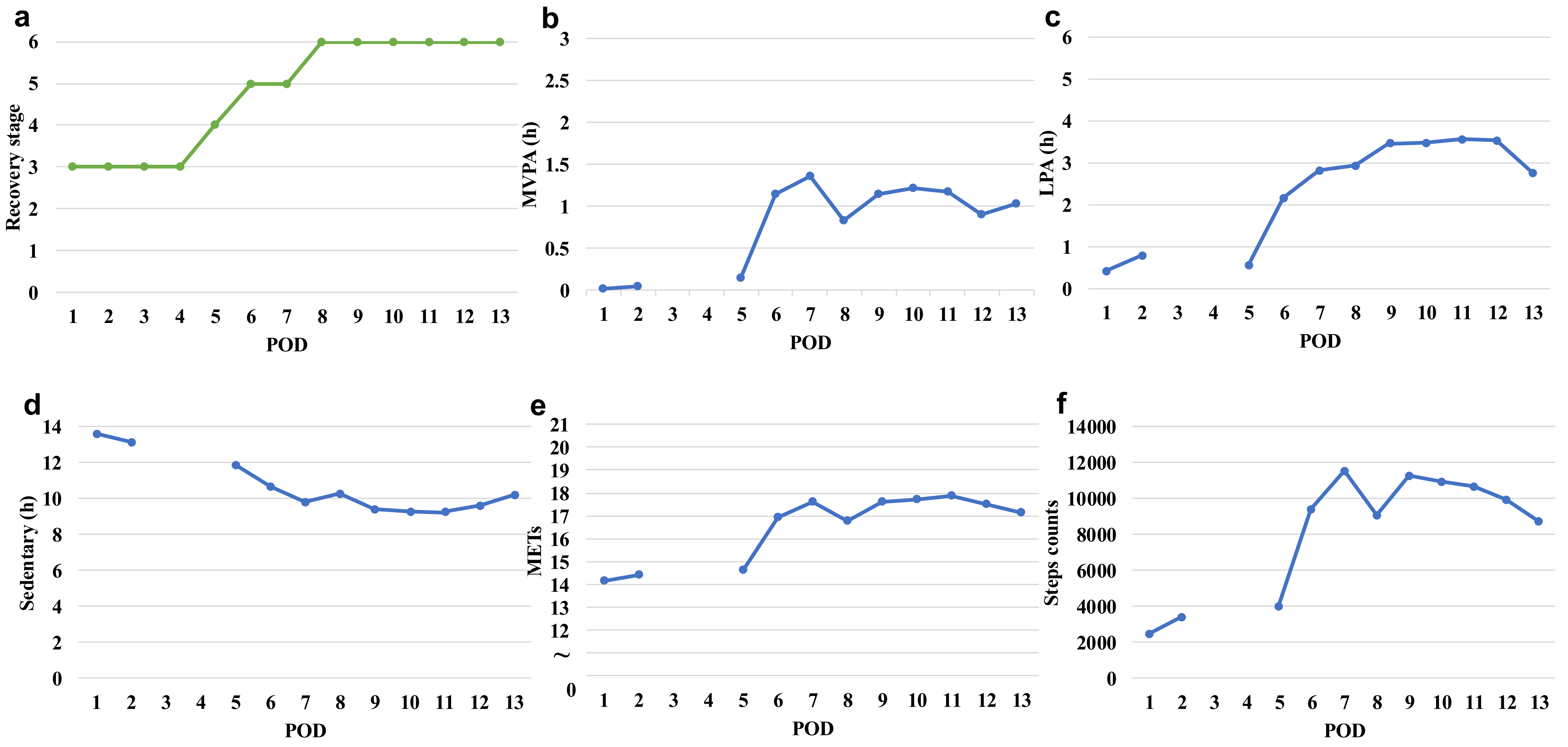

AC-09: non-smooth recovery

T. Yamane, Y. Kimura, M. Tetsutani, et al., "Indicators for Monitoring Recovery From Surgery to Discharge Using Accelerometer in Patients With Esophageal Cancer," *World Journal of Surgery* (2026): https://doi.org/10.1002/wjs.70472

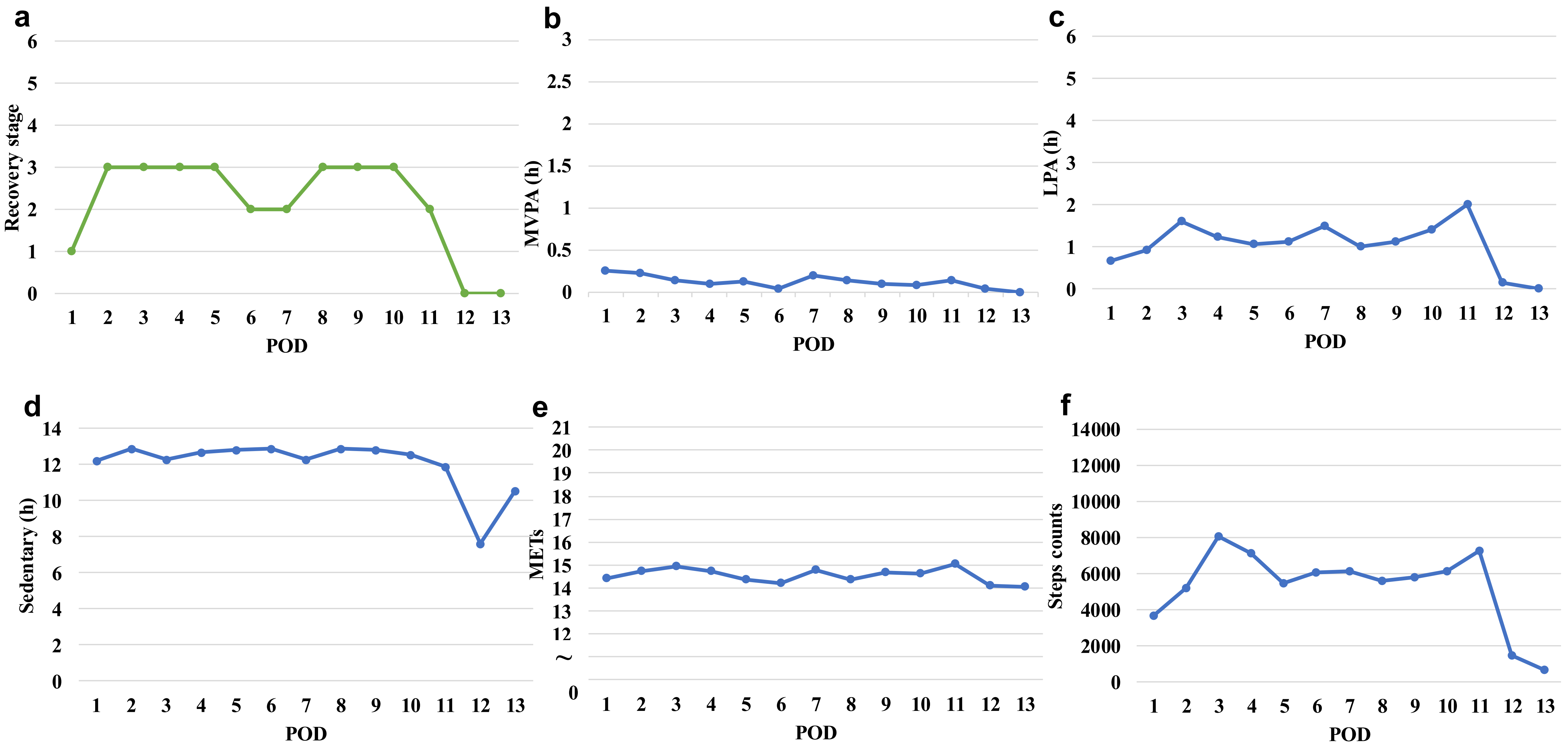

AC-10: non-smooth recovery

T. Yamane, Y. Kimura, M. Tetsutani, et al., "Indicators for Monitoring Recovery From Surgery to Discharge Using Accelerometer in Patients With Esophageal Cancer," *World Journal of Surgery* (2026): https://doi.org/10.1002/wjs.70472

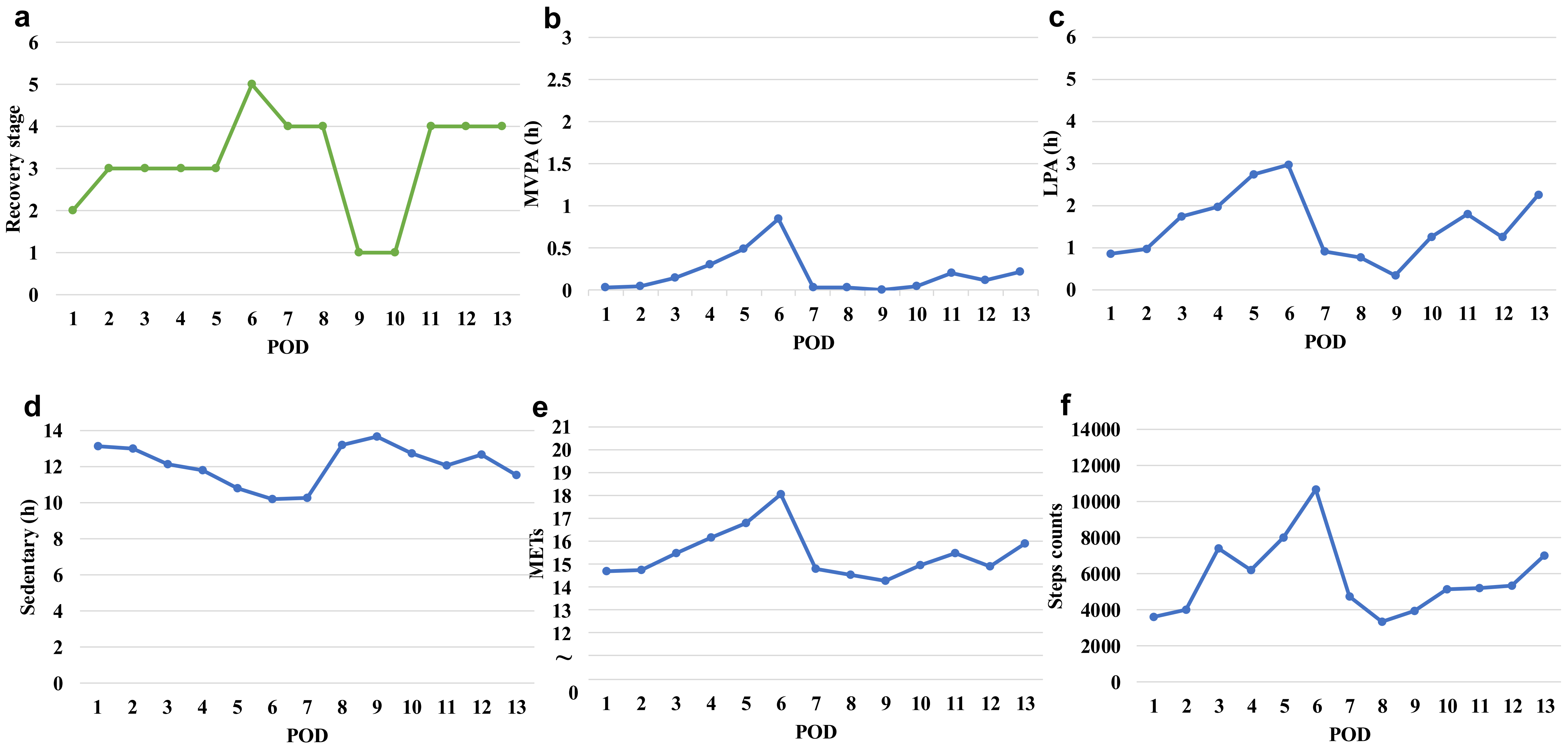

AC-11: smooth recovery

T. Yamane, Y. Kimura, M. Tetsutani, et al., "Indicators for Monitoring Recovery From Surgery to Discharge Using Accelerometer in Patients With Esophageal Cancer," *World Journal of Surgery* (2026): https://doi.org/10.1002/wjs.70472

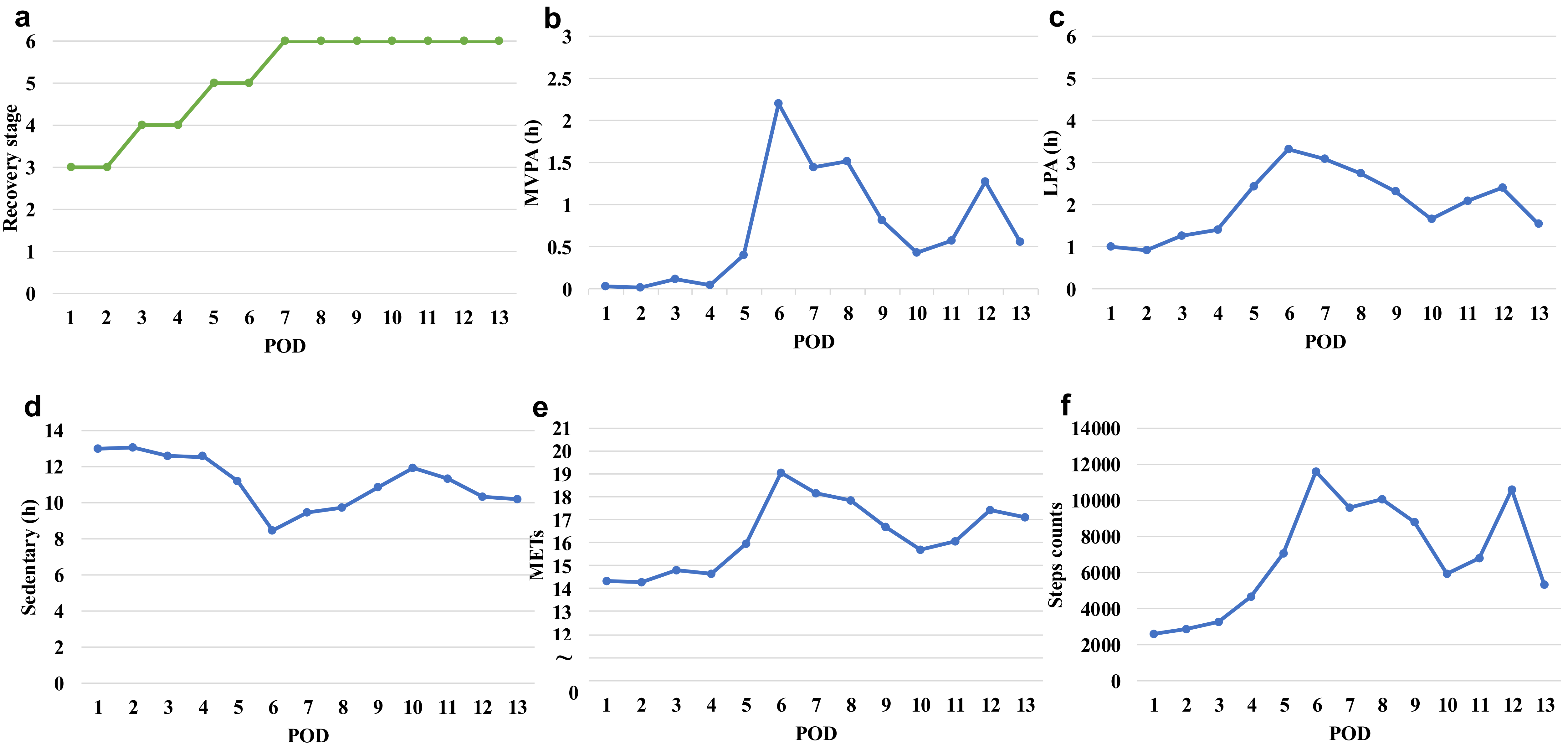

AC-12: smooth recovery

T. Yamane, Y. Kimura, M. Tetsutani, et al., "Indicators for Monitoring Recovery From Surgery to Discharge Using Accelerometer in Patients With Esophageal Cancer," *World Journal of Surgery* (2026): https://doi.org/10.1002/wjs.70472

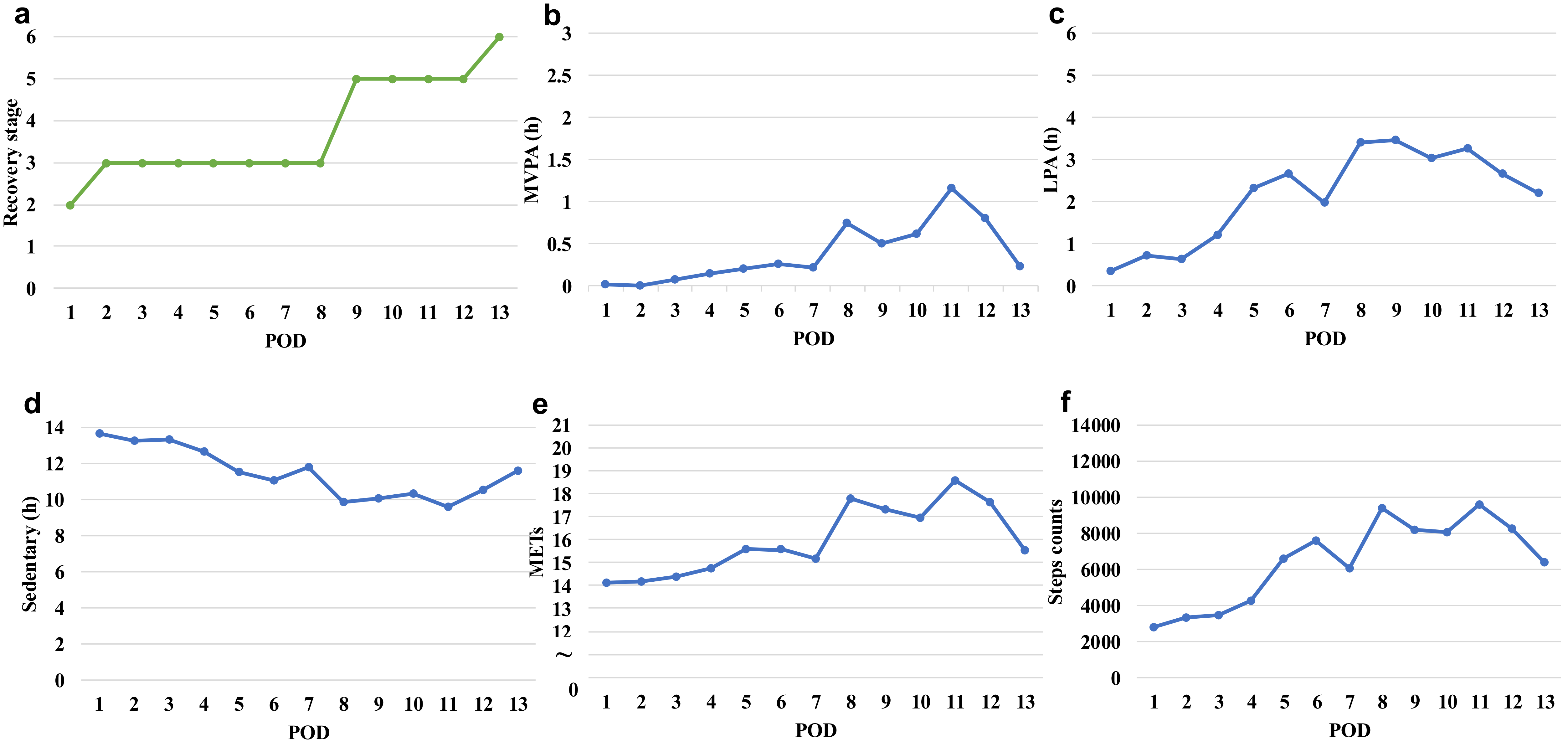

AC-13: smooth recovery

T. Yamane, Y. Kimura, M. Tetsutani, et al., "Indicators for Monitoring Recovery From Surgery to Discharge Using Accelerometer in Patients With Esophageal Cancer," *World Journal of Surgery* (2026): https://doi.org/10.1002/wjs.70472

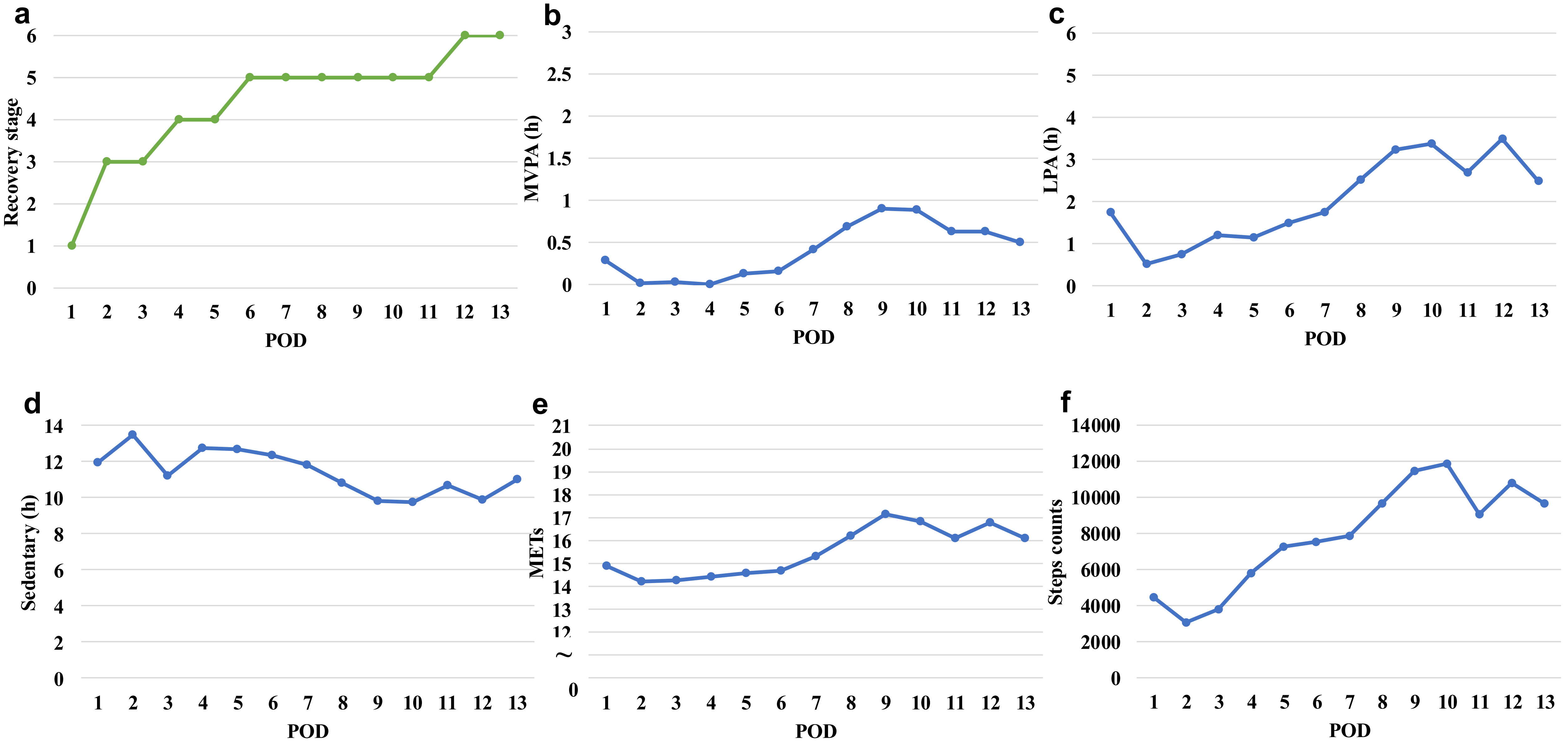

AC-14: smooth recovery

T. Yamane, Y. Kimura, M. Tetsutani, et al., "Indicators for Monitoring Recovery From Surgery to Discharge Using Accelerometer in Patients With Esophageal Cancer," *World Journal of Surgery* (2026): https://doi.org/10.1002/wjs.70472

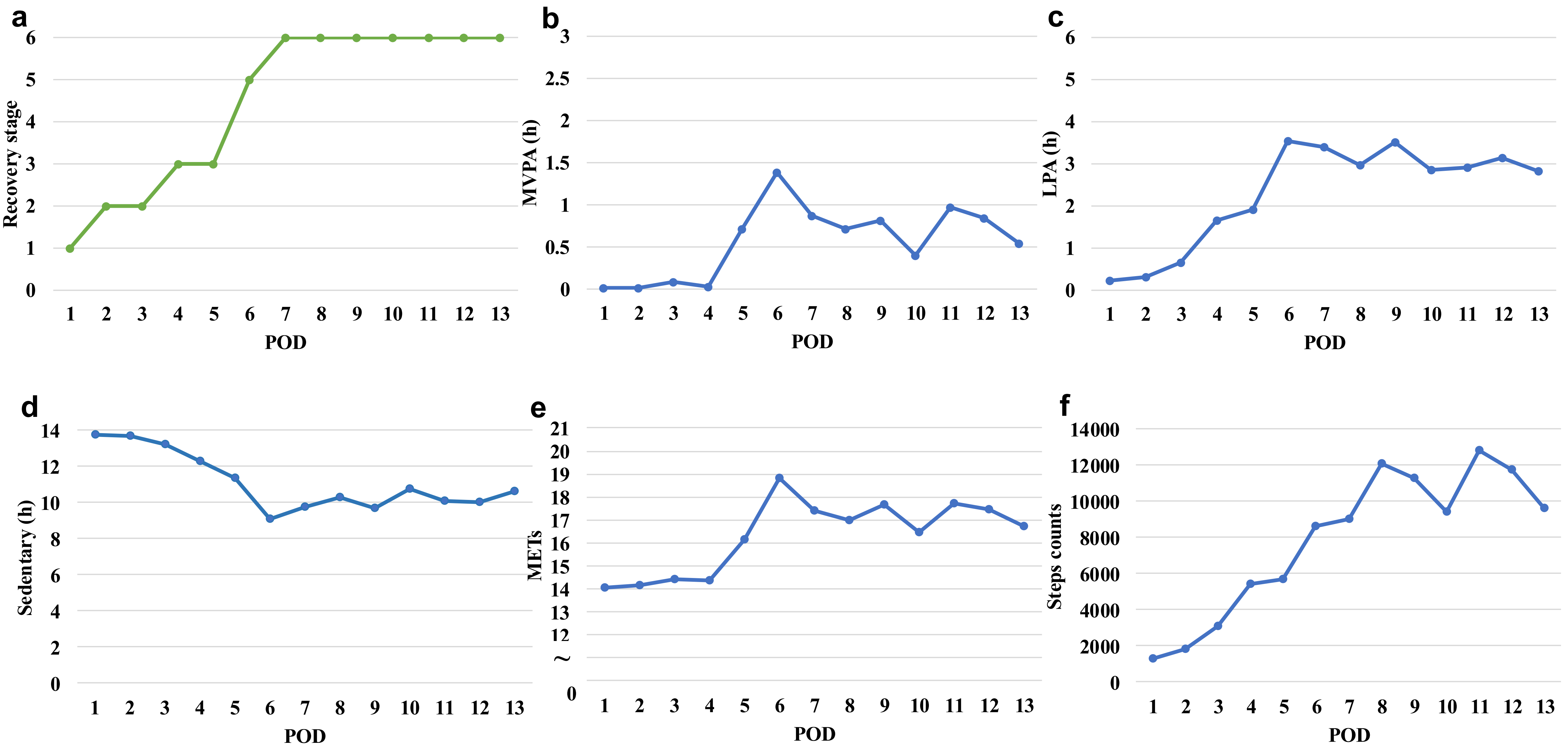

AC-15: smooth recovery

T. Yamane, Y. Kimura, M. Tetsutani, et al., "Indicators for Monitoring Recovery From Surgery to Discharge Using Accelerometer in Patients With Esophageal Cancer," *World Journal of Surgery* (2026): https://doi.org/10.1002/wjs.70472

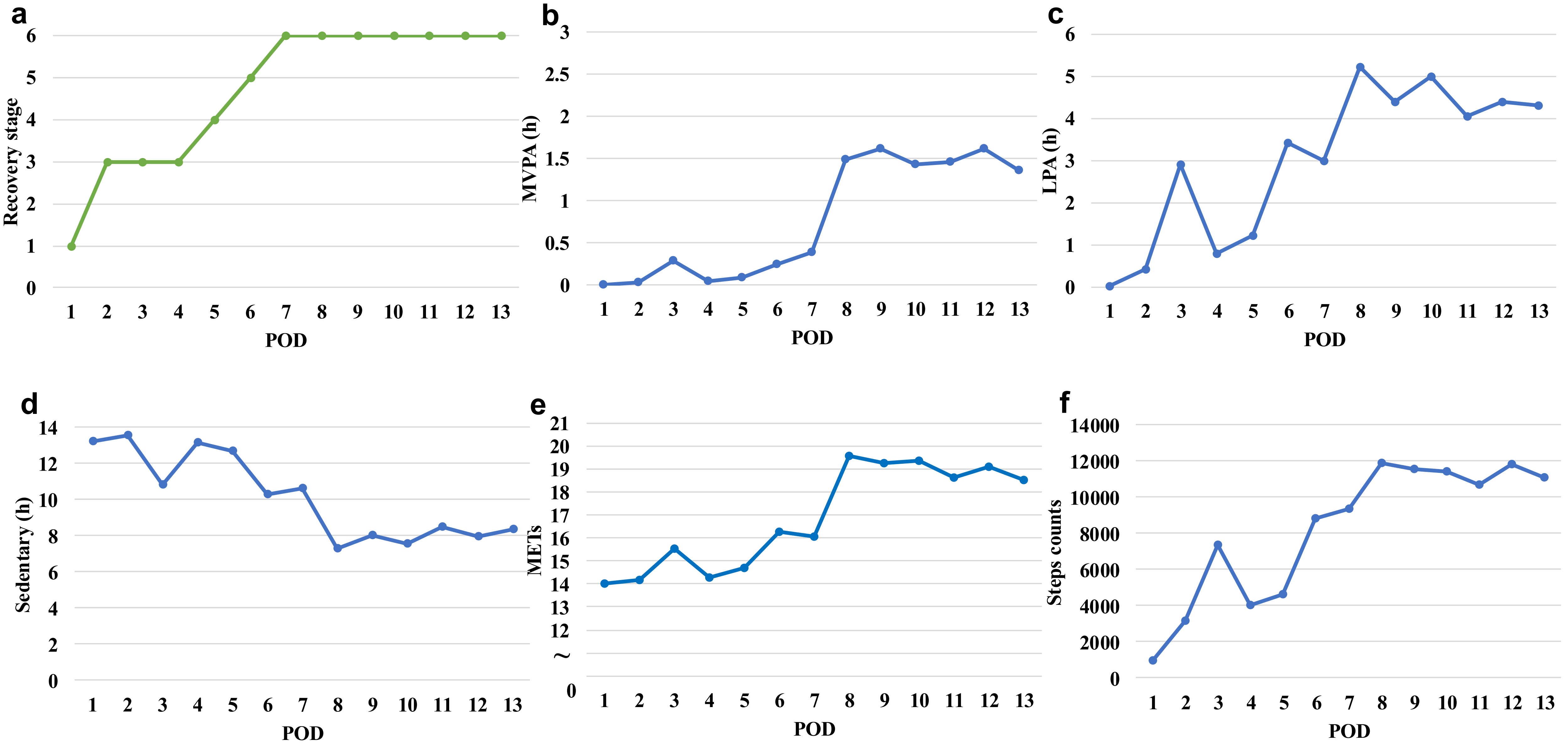

T. Yamane, Y. Kimura, M. Tetsutani, et al., “Indicators for Monitoring Recovery From Surgery to Discharge Using Accelerometer in Patients With Esophageal Cancer,” *World Journal of Surgery* (2026): https://doi.org/10.1002/wjs.70472

AC-17: non-smooth recovery

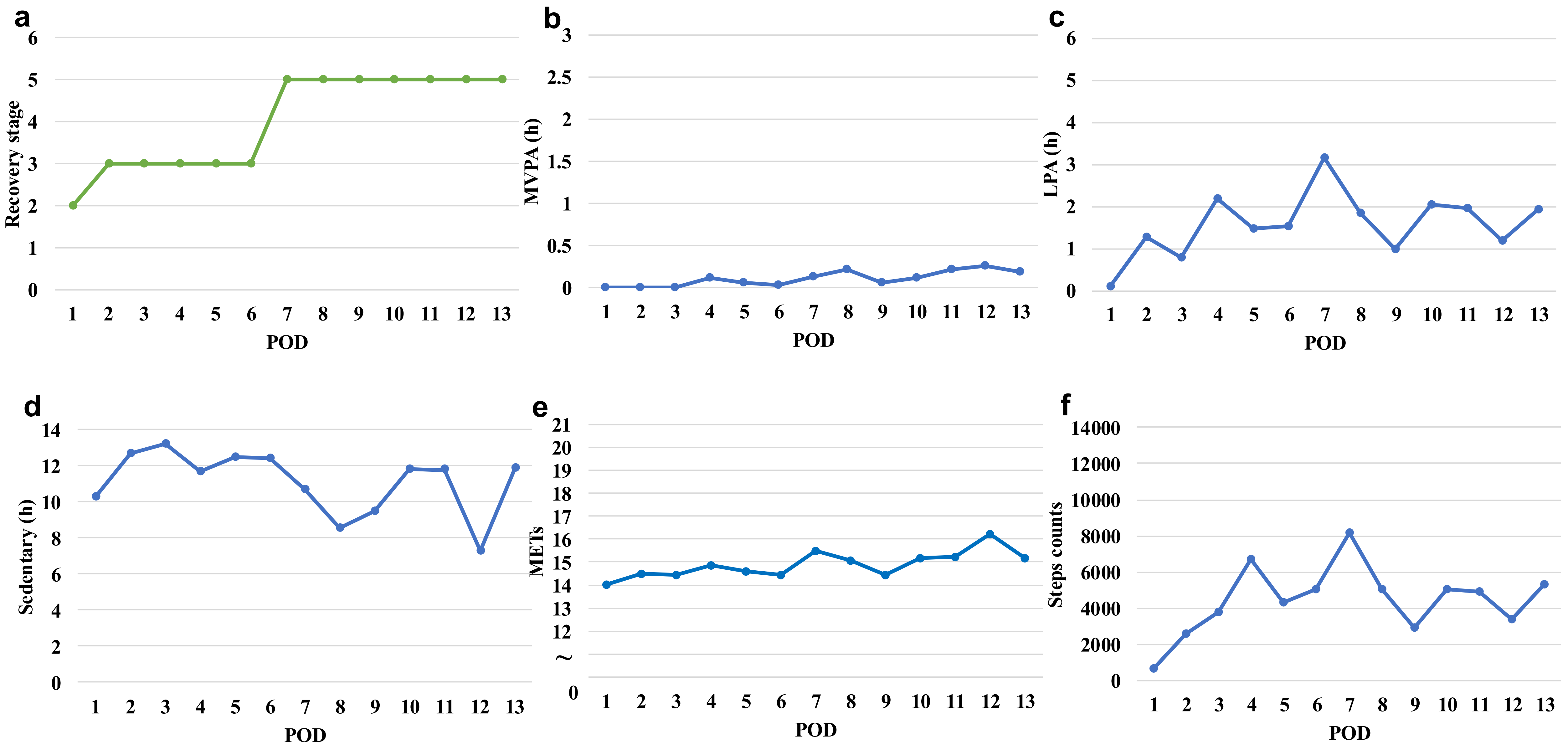

AC-18: non-smooth recovery

T. Yamane, Y. Kimura, M. Tetsutani, et al., "Indicators for Monitoring Recovery From Surgery to Discharge Using Accelerometer in Patients With Esophageal Cancer," *World Journal of Surgery* (2026): https://doi.org/10.1002/wjs.70472

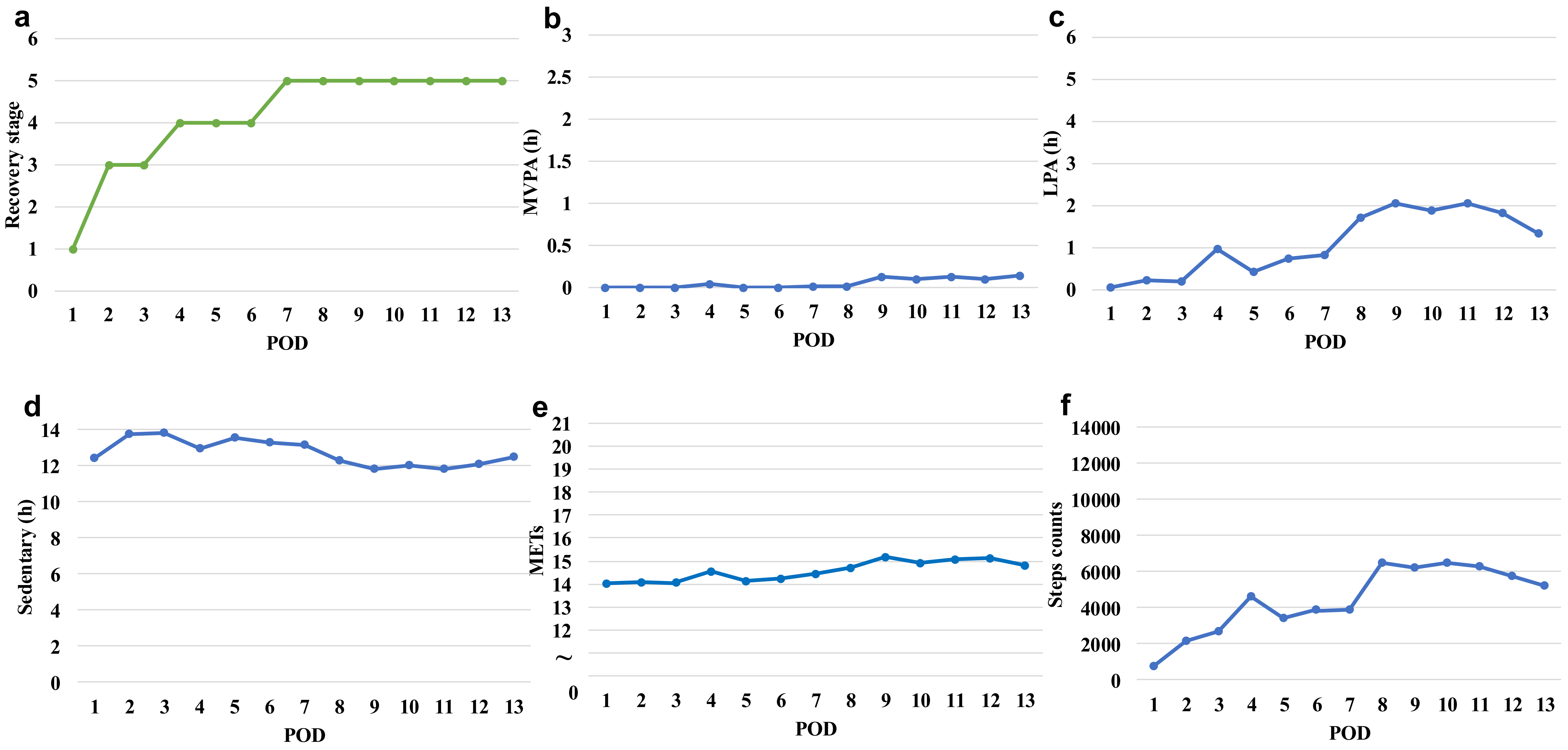

AC-19: smooth recovery

T. Yamane, Y. Kimura, M. Tetsutani, et al., "Indicators for Monitoring Recovery From Surgery to Discharge Using Accelerometer in Patients With Esophageal Cancer," *World Journal of Surgery* (2026): https://doi.org/10.1002/wjs.70472

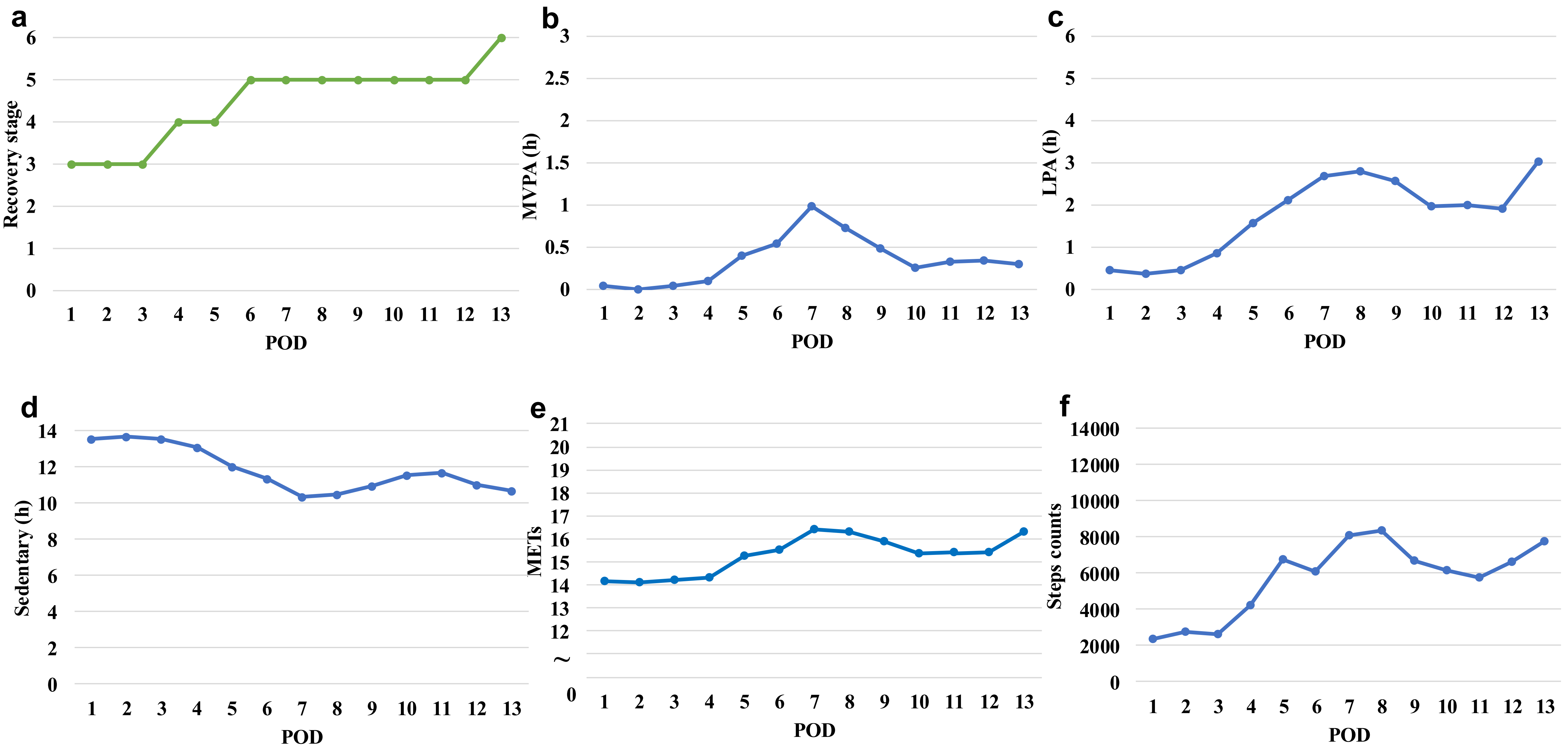

AC-20: non-smooth recovery

T. Yamane, Y. Kimura, M. Tetsutani, et al., "Indicators for Monitoring Recovery From Surgery to Discharge Using Accelerometer in Patients With Esophageal Cancer," *World Journal of Surgery* (2026): https://doi.org/10.1002/wjs.70472

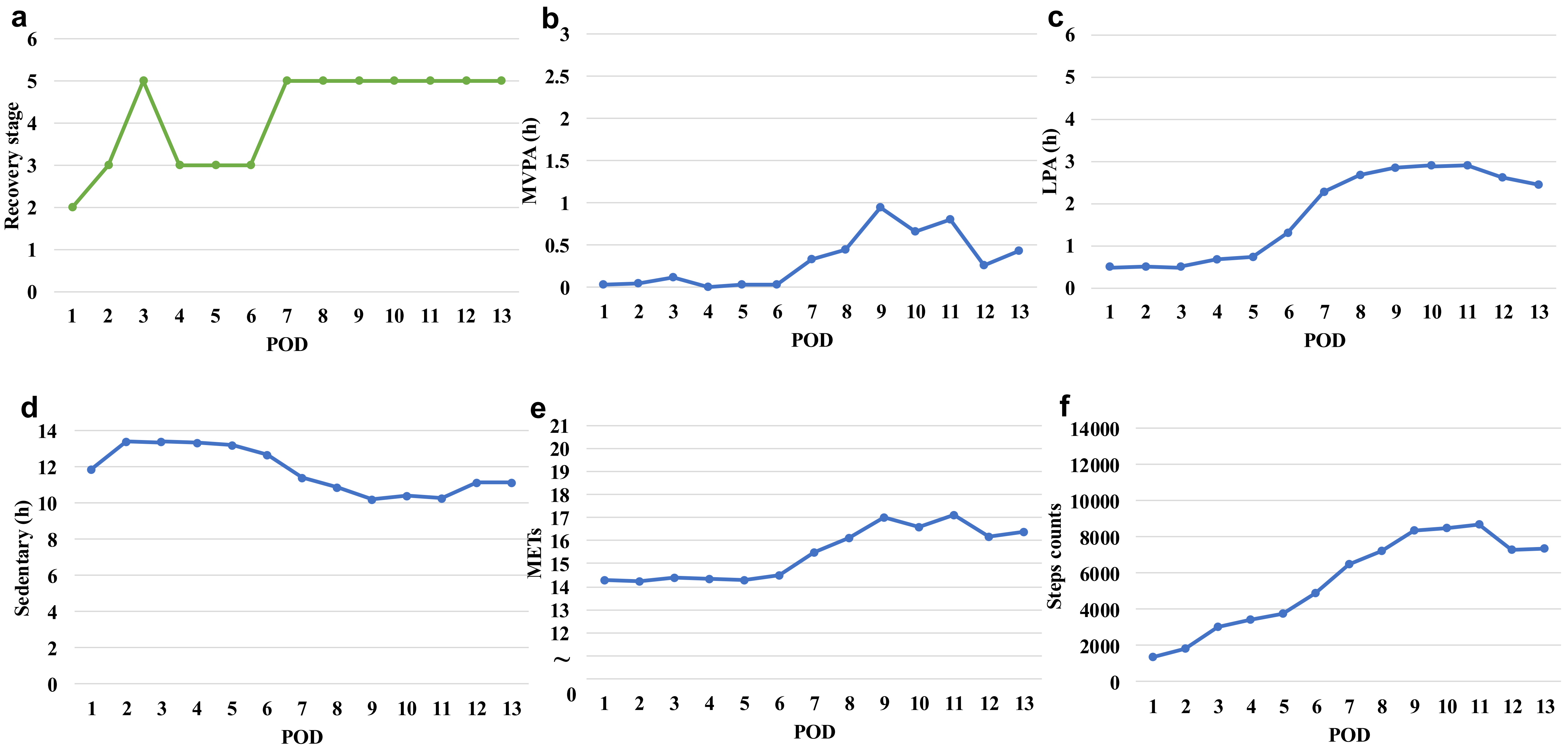

AC-21: non-smooth recovery

T. Yamane, Y. Kimura, M. Tetsutani, et al., "Indicators for Monitoring Recovery From Surgery to Discharge Using Accelerometer in Patients With Esophageal Cancer," *World Journal of Surgery* (2026): https://doi.org/10.1002/wjs.70472

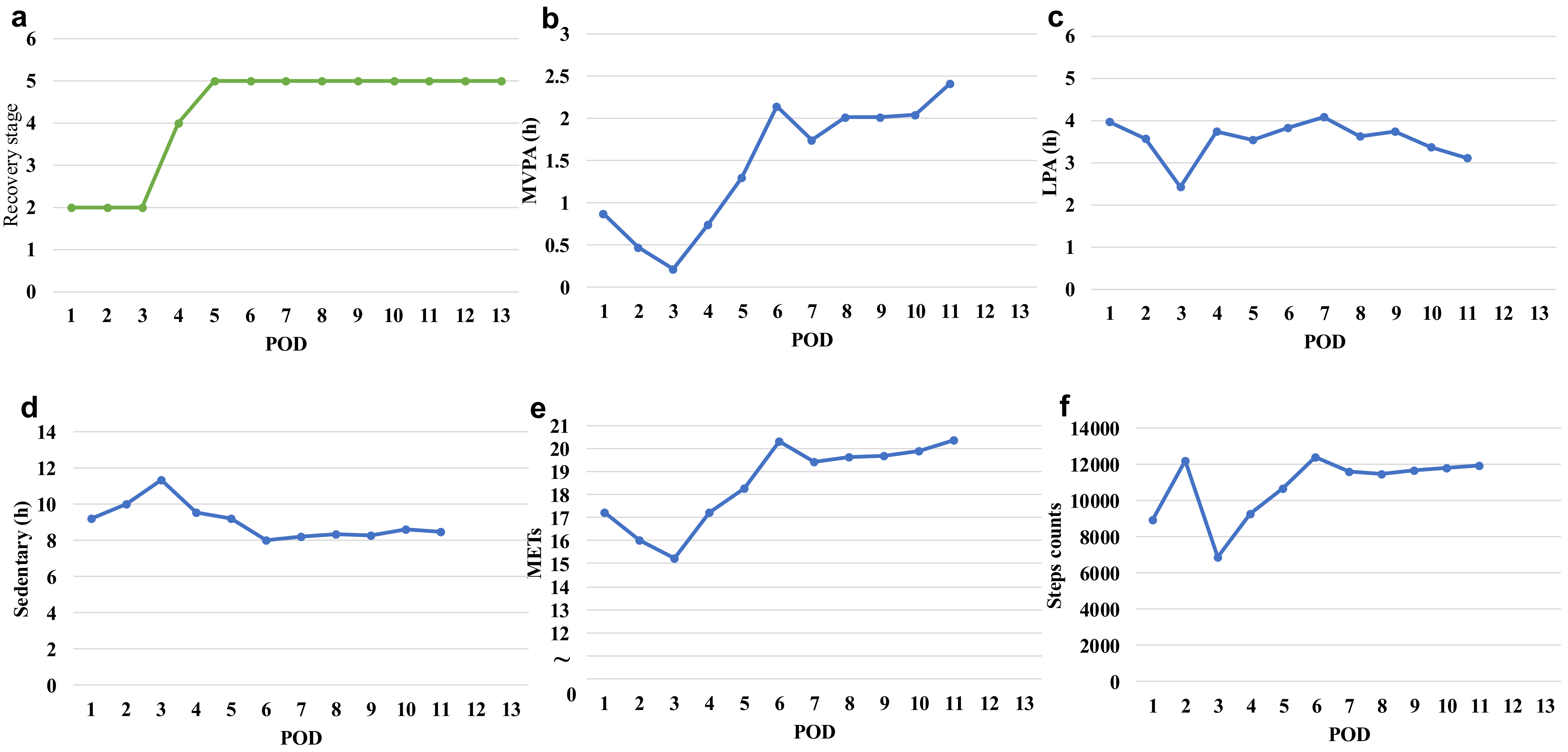

Supplement: Supplementary file 7 [file WJS-50-2434-s007.pdf]
